# A Novel Nanoproteomic Approach for the Identification of Molecular Targets Associated with Thyroid Tumors

**DOI:** 10.3390/nano10122370

**Published:** 2020-11-28

**Authors:** María García-Vence, María del Pilar Chantada-Vázquez, José Manuel Cameselle-Teijeiro, Susana B. Bravo, Cristina Núñez

**Affiliations:** 1 Proteomic Unit, Instituto de Investigaciones Sanitarias, Complejo Hospitalario Universitario de Santiago de Compostela (IDIS-CHUS), 15706 Santiago de Compostela, Spain; mariagarve@outlook.es (M.G.-V.); mariadelpilarchantadavazquez@gmail.com (M.d.P.C.-V.); 2 Research Unit, Hospital Universitario Lucus Augusti (HULA), Servizo Galego de Saúde (SERGAS), 27002 Lugo, Spain; 3 Department of Anatomic Pathology, Clinical University Hospital, Instituto de Investigación Sanitaria (IDIS), Galician Healthcare Service (SERGAS), 15703 Santiago de Compostela, Spain; 4 School of Medicine, University of Santiago de Compostela, 15706 Santiago de Compostela, Spain

**Keywords:** gold nanoparticles (AuNPs), silver nanoparticles (AgNPs), magnetic nanoparticles (FeNPs), protein corona (PC), thyroid cancer, molecular target

## Abstract

A thyroid nodule is the most common presentation of thyroid cancer; thus, it is extremely important to differentiate benign from malignant nodules. Within malignant lesions, classification of a thyroid tumor is the primary step in the assessment of the prognosis and selection of treatment. Currently, fine-needle aspiration biopsy (FNAB) is the preoperative test most commonly used for the initial thyroid nodule diagnosis. However, due to some limitations of FNAB, different high-throughput “omics” approaches have emerged that could further support diagnosis based on histopathological patterns. In the present work, formalin-fixed paraffin-embedded (FFPE) tissue specimens from normal (non-neoplastic) thyroid (normal controls (NCs)), benign tumors (follicular thyroid adenomas (FTAs)), and some common types of well-differentiated thyroid carcinoma (follicular thyroid carcinomas (FTCs), conventional or classical papillary thyroid carcinomas (CV-PTCs), and the follicular variant of papillary thyroid carcinomas (FV-PTCs)) were analyzed. For the first time, FFPE thyroid samples were deparaffinized using an easy, fast, and non-toxic method. Protein extracts from thyroid tissue samples were analyzed using a nanoparticle-assisted proteomics approach combined with shotgun LC-MS/MS. The differentially regulated proteins found to be specific for the FTA, FTC, CV-PTC, and FV-PTC subtypes were analyzed with the bioinformatic tools STRING and PANTHER showing a profile of proteins implicated in the thyroid cancer metabolic reprogramming, cancer progression, and metastasis. These proteins represent a new source of potential molecular targets related to thyroid tumors.

## 1. Introduction

Thyroid cancer, the most common endocrine malignancy, rises by half a million new cases worldwide each year [1]. A thyroid nodule, the most common presentation of thyroid cancer, might be due to multiple causes, making it important to differentiate benign from malignant nodules [2].

Within malignant lesions, the classification of thyroid tumors is the first step in the assessment of the prognosis and treatment decisions [3]. Thyroid malignancy includes follicular-cell derived and C-cell derived tumors. Follicular tumors can be classified as (a) well-differentiated (papillary thyroid carcinoma (PTC), follicular thyroid carcinoma (FTC), and Hürthle (oncocytic) cell carcinoma (HCC)), (b) poorly differentiated thyroid carcinoma (PDTC), and (c) anaplastic (undifferentiated) thyroid carcinoma (ATC). While benign follicular cell-derived tumors are called follicular thyroid adenoma (FTA) or oncocytic thyroid adenoma (OTA), all C-cell derived tumors are malignant and called medullary thyroid carcinoma (MTC) [3].

Currently, fine-needle aspiration biopsy (FNAB) is the preoperative test most commonly used for the initial thyroid nodule diagnosis [4]. In some cases, however, the cytological and histological classification of thyroid tumors is problematic [5]. Some variants of PTC share several cytological features with benign lesions (hyperplastic nodules, FTA, Hashimoto’s thyroiditis, etc.). Cytological differentiation between PTC and other malignant lesions (HCC, FTC, and MTC) can also be problematic [6,7,8,9]. FNAB cannot differentiate between FTA and FTC or between OTA and HCC; these cytological samples are usually diagnosed as indeterminate or follicular-patterned thyroid lesions [7,10,11,12,13]. For this reason, histopathological examination of the surgical specimen is mandatory to determine whether there is capsular and/or vascular invasion (malignant) or not (benign) [3,13]. The necessity of a hemithyroidectomy alone or a total thyroidectomy, as well as additional radioiodine therapy is then determined based on the degree of malignancy and/or risk factors [10,11]. Only 8–17% of these cytologically suspicious nodules result in being malignant upon histology [12], meaning that many patients are still subjected to unnecessary surgery. Therefore, it is of prime importance to develop better preoperative diagnosis methods of malignant thyroid neoplasms [13].

Although the diagnosis of PTC could be confirmed by several candidate biomarkers, they have not been implemented in clinical practice [14]. Similarly, the biomarkers used to discriminate between FTA and FTC have not been validated, histological evaluation being the gold standard for the distinction between these lesions [15].

The identification of novel biomarkers through high-throughput “omics” methods maintains the diagnosis of thyroid cancers based on histopathological patterns, improving their classification [16,17,18,19,20,21]. Although most of the studies are based on gene expression profiling and mutations [22,23,24,25], some proteomics studies have also identified potential biomarker proteins for the discrimination between cancerous and normal thyroid tissue [26,27], malignant and benign follicular lesions [28,29,30], or papillary and follicular cancers [28,31].

FNAB samples [32,33,34], fresh and frozen thyroid tumor samples [24,25,26,27,28,29], and cell lines [35,36] are the kind of samples most commonly used for the development of these proteomic studies in thyroid malignancies, but the use of formalin-fixed paraffin-embedded (FFPE) thyroid samples has been less widely extended [37,38,39]. Although the FFPE method is often used for long-term tissue preservation, it causes the formation of protein cross-links that make protein extraction more difficult [40,41,42,43,44,45]. Despite these drawbacks, many articles describe the use of proteins extracts from FFPE samples [46,47,48,49,50], and particularly from FFPE thyroid samples [35].

Recently, a new research method named nanoproteomics has emerged as a result of the introduction of nanomaterials into the field of proteomics [51]. When nanoparticles (NPs) are exposed to biological fluids and cell and tissue lysates, a biological complex around the NPs is formed named protein corona (PC) [52]. The PC composition could be affected by multiple factors: the characteristics of the NPs, such as size, charge, and surface functionalization; the composition of the NP core; the characteristics of the protein as isoelectric points, molecular weight, structure, and folding; the characteristics of the interaction conditions, such as temperature, time, and concentration; and the type of biological sample, which could be multiple types (sera, plasma, urine, tissue lysate, cell lysate, etc.) [50].

Recently, several works described that NP surfaces can sequester and enrich different proteins that were identified as novel disease-based therapeutic targets [53]. The characterization of the PC around NPs offers distinct advantages over sole proteomic approaches and increases the success of identifying molecular targets [54]. Thus, the identification of proteins in the PC of NPs from healthy and pathological samples (serum/plasma samples [55,56,57,58], tissues lysates [59], and cell lysates [60]) together with comparative proteomic studies and bioinformatic analyses provides the identification of novel molecular targets.

In this way, different authors have identified novel molecular targets after the analysis of the PC formed around AuNPs (with a high surface area) [56,57,58,61], AgNPs (with a strong antibacterial activity) [56,62], and FeNPs (with magnetic properties, low toxicity, and biodegradability) [57,63] in contact with different biological media.

In the present work, for the first time, we develop a nanoparticle-assisted proteomics approach based on AuNP-, AgNP-, and FeNP-protein corona fingerprinting combined with shotgun LC-MS/MS analysis for the identification of novel molecular targets in protein extracts from FFPE tissue samples of different types of thyroid cancer.

## 2. Materials and Methods

### 2.1. Reagents

All reagents and solvents used were HPLC or LC-MS grade. Ammonium bicarbonate (AMBIC) (NH_4_HCO_3_, 99.5%, ammonium hydroxide (NH_4_OH), ammonium persulfate (APS) ((NH_4_)_2_S_2_O_8_, 98%), β-mercaptoethanol (C_2_H_6_OS), 3-[(3-cholamidopropyl)dimethylammonio]-1-propanesulfonate hydrate (CHAPS) (C_32_H_58_N_2_O_7_S·xH_2_O), ethylenediaminetetraacetic acid (EDTA) ((HO_2_CCH_2_)_2_NCH_2_CH_2_N(CH_2_CO_2_H)_2_, 99%), formaldehyde (HCHO, 36.5–38% in H_2_O), glycerol (HOCH_2_CH(OH)CH_2_OH, 99%), iron(III) chloride hexahydrate (FeCl_3_·6H_2_O, 97%), iron(II) sulfate heptahydrate (FeO_4_S·7H_2_O, 99%), silver nitrate (AgNO_3_, 99%), sodium borohydride (NaBH_4_, 98%), sodium carbonate (Na_2_CO_3_, 99.0%), sodium citrate tribasic dihydrate (HOC(COONa)(CH_2_COONa)_2_·2H_2_O, 99%), sodium chloride (NaCl, 99.5%), sodium deoxycholate (C_24_H_39_NaO_4_, 97%), tannic acid (C_76_H_52_O_46_), thiourea (NH_2_CSNH_2_, 99%), tributylphosphine ([CH_3_(CH_2_)_3_]_3_P, 97%), trifluoroacetic acid (TFA) (CF_3_COOH, 99%), tris-base (NH_2_C(CH_2_OH)_3_), triton X-100 (*t*-Oct-C_6_H_4_-(OCH_2_CH_2_)_x_OH), trizma base (NH_2_C(CH_2_OH)_3_, 99.9%), urea (NH_2_CONH_2_), and cOmplete™ ULTRA Tablets, Mini, EASYpack Protease Inhibitor Cocktail. were purchased from Sigma-Aldrich (St. Louis, MO, USA). Acrylamide/bis-acrylamide 30% solution (37.5:1) was purchased from SERVA Electrophoresis GmbH (Heidelberg, Germany). Trypsin sequence-grade was purchased from Promega (USA). Bromophenol-blue, Coomassie Brilliant Blue R250 staining solution, 1,4-dithiothreitol (DTT), iodoacetamide (IAA), sodium dodecyl sulphate (SDS), *N*,*N*,*N’*,*N’*-tetramethylethylene-diamine (TEMED), and the molecular scale marker (wide range, mol wt 6.5-200 kDa) for gel electrophoresis were purchased from Bio-Rad (Madrid, Spain). Hydrogen tetrachloroaurate (III) hydrate 99.9% (metals basis), Au 49% min (HAuCl_4_·xH_2_O), was purchased from Strem Chemicals (Massachusetts, USA). Acetonitrile (ACN), ethanol (EtOH), formic acid (HCOOH), glacial acetic acid, hydrochloric acid (HCl), isobutanol, and methanol (MeOH) were supplied by *Panreac Química SLU* (Barcelona, Spain).

### 2.2. Synthesis and Characterization of AgNPs (9.66 ± 1.77), AuNPs (7.55 ± 0.70), and FeNPs (8.25 ± 0.78)

**Figure 1:**
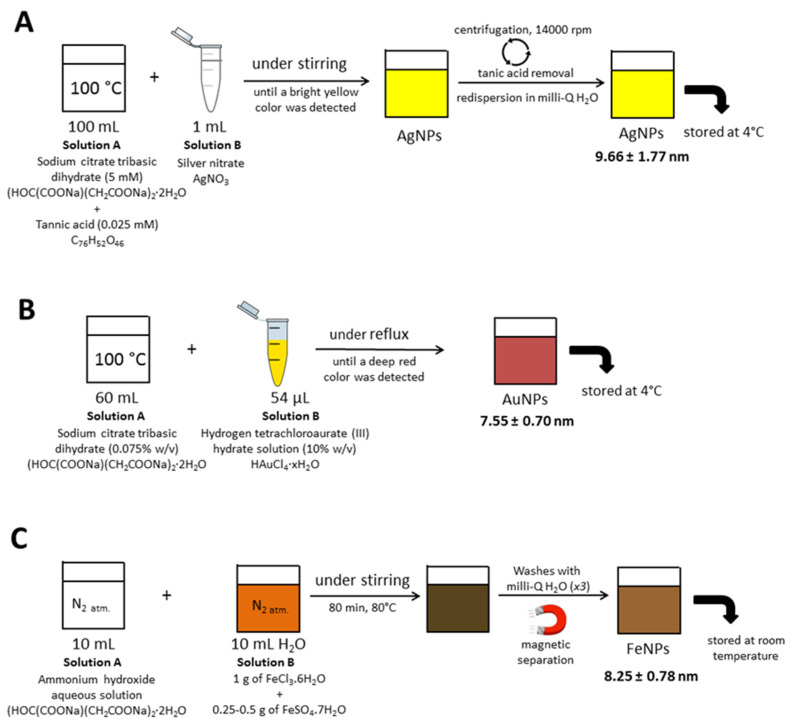
Schematic representation for the synthesis of (**A**) AgNPs (9.66 ± 1.77), (**B**) AuNPs (7.55 ± 0.70), and (**C**) FeNPs (8.25 ± 0.78).

Synthesis of AgNPs (9.66 ± 1.77), AuNPs (7.55 ± 0.70), and FeNPs (8.25 ± 0.78) was performed according to a modification of a previously reported method illustrated in Figure 1 [57]. Transmission electron microscopy (TEM) characterizations of AuNPs, AgNPs, and FeNPs were performed using a Jeol JEM 1011 microscope (JEOL, Madrid, Spain).

### 2.3. Tissue Specimens

All thyroid samples incorporated in this study belong to the Biobank of the Clinical University Hospital of Santiago de Compostela (CHUS), Spain (PT17/0015/0002), integrated into the Spanish National Biobank Network (“TIROCHUS collection” with code C.0003960). The study was conducted in conformity with the declaration of Helsinki and approved by the Clinical Research Ethics Committees (CEIC) of Galicia (Spain) with Approval Number 2016/239. All participants from CHUS (Spain) gave written informed consent before their participation.

Thyroid tissue samples were obtained from four cases of FTA, five cases of FTC, five cases of CV-PTC, three cases of FV-PTC, and five cases of normal (non-neoplastic) thyroid tissue (normal controls (NCs)).

All thyroid tissue samples were obtained from total or partial thyroidectomy specimens, which had been FFPE, following the routine procedures in the laboratory of the Department of Pathology of the CHUS.

### 2.4. Deparaffinization of FFPE Tissue Blocks with Hot Water and Protein Extraction

Paraffin from the thyroid tissue samples was removed following the method of Mansour et al. [64], with some minor variations. Briefly, FFPE thyroid tissue sections (2–3 sections of 10 µm thick) were placed in Eppendorf tubes and deparaffinized by incubating them three times with milli-Q water (400 µL) preheated at 90–95 °C for 2–3 min in a Thermomixer (Eppendorf, Hamburg, Germany) at 500–600 rpm, fixing the temperature at 95 °C. After that, the samples were resuspended in milli-Q water at room temperature and stored at −20 °C (ca. 12 h) until the protein extraction step.

For the protein extraction process, water from the frozen thyroid tissue sections was eliminated, and 150 µL of a buffer solution were added to each sample. The buffer composition was 20 mM Tris-HCl pH 8.8, 2% SDS, 1% CHAPS, 200 mM DTT, 200 mM glycine, and a mixture of protease inhibitors [65]. After that, samples were heated at 100 °C, for 20 min and then at 60 °C for 2 h. Later, the samples were sonicated in a water bath sonicator (Branson Ultrasonics, Danbury, CT, USA) for 30 min. Samples were then stored at −20 °C until the next day. After the addition of 50 µL of the buffer, each sample was treated 3 min with a TissueLyser (Qiagen, Madrid, Spain) to improve tissue fragmentation. Then, samples were centrifuged at 14,000 rpm for 20 min to remove tissue remnants (pellets). Protein extracts (supernatants) were transferred to clean Eppendorf tubes and stored at −20 °C until further use.

### 2.5. Ex Vivo Protein Corona Formation: Interaction of Proteins Presented in Tissue Extracts with AgNPs (9.66 ± 1.77 nm), AuNPs (7.55 ± 0.70 nm), and FeNPs (8.25 ± 0.78 nm)

**Figure 2:**
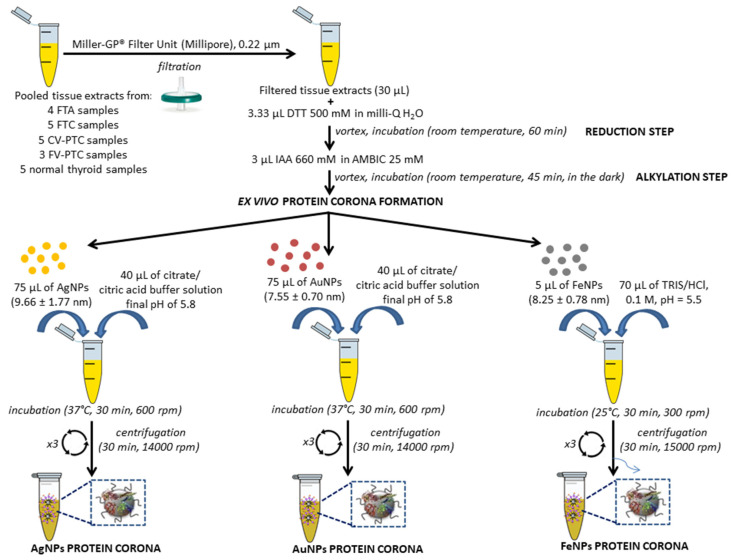
Flowchart depicting tissues extract pre-treatment and AgNP-, AuNP-, and FeNP-protein corona formation. FTA, follicular thyroid adenoma; FTC, follicular thyroid carcinoma; CV-PTC, conventional or classical papillary thyroid carcinoma; DTT, 1,4-dithiothreitol; IAA, iodoacetamide.

Five pools were prepared from the tissue extracts of four FTAs, five FTCs, five CV-PTCs, three FV-PTCs, and five normal thyroid tissue samples (controls). All pooled tissue extracts were treated following our previously reported method for the PC formation in serum, with some modifications [56,57,58] (see Figure 2).

Briefly, after the reduction (with DTT) and the alkylation (with IAA) of proteins present in the tissue extracts (30 μL), seventy-five microliters of AgNPs (9.66 ± 1.77 nm), 75 μL of AuNPs (7.55 ± 0.70 nm), and 5 μL of FeNPs (8.25 ± 0.78 nm) were added to each protein extract (×2), maintaining NP/protein ratios of 1:2 and pH values of 5.8 (AgNPs and AuNPs) and 5.1 (MNPs). While AgNP- and AuNP-tissue extracts were incubated at 37 °C in a thermostatic bath for 30 min with shaking (600 rpm), FeNP-tissue extracts were incubated at 25 °C for 30 min (300 rpm).

Pellets were harvested by centrifugation at 14,000 rpm (AgNPs and AuNPs) and 15,000 rpm (FeNPs) for 30 min. Pellets containing PC-AgNPs and PC-AuNPs were washed (×3) with 100 μL citrate/citric acid buffer (pH 5.8) and centrifugated at 14,000 rpm (AgNPs and AuNPs). Fifty microliters of TRIS-HCl 0.1 M (pH 5.5) were used to wash (×2) the pellets of PC-FeNPs, and after that, they were centrifuged at 15,000 rpm for 30 min to remove proteins unbound to the nanoparticles’ surface.

### 2.6. Separation of Corona Proteins by SDS-PAGE, In-Gel Protein Digestion, and Identification by Mass Spectrometry

Corona proteins associated with AgNPs (9.66 ± 1.77 nm), AuNPs (7.55 ± 0.70 nm), and FeNPs (8.25 ± 0.78 nm) were separated by SDS-PAGE (×2) in a Power Pac Basic power supply from Bio-Rad (Hercules, CA, USA) and digested following the scheme represented in Figure S1 (Supplementary Materials).

The digestion was stopped with the addition of 50 μL of 5% (*v*/*v*) formic acid. After that, the extraction of the peptides from the gel was carried out with a solution of 50% (*v*/*v*) ACN/0.1% (*v*/*v*) TFA (×3) and ACN (×1). Samples were dried and stored at −20 °C until further use [66].

Digested peptides of each sample were separated using reverse phase chromatography (×3). Mass spectrometry was applied accordingly to our previously published protocol [56] using an Eksigent Technologies nanoLC 400 (ABSciex, Madrid, Spain) (a micro liquid chromatography system), coupled to a high speed TripleTOF 6600 mass spectrometer with a microflow source.

The analytical and the trap columns used were a silica-based reverse phase column Eksigent C18CL (Eksigent, ABSciex, Madrid, Spain) and a YMC-TRIART C18, respectively. Both columns operated on-line and presented 3 mm particle size and 120 Å pore size. The loading pump and the micro-pump provided flowrates of 10 μL/min and 5 μL/min, respectively. The injection volume was fixed at 4 μL.

A TripleTOF 6600 System (SCIEX, Foster City, CA, USA) was employed for the data acquisition using a data-dependent workflow. After MS/MS analysis, data files were processed using ProteinPilot^TM^ 5.0.1 software from ABSciex (Madrid, Spain), which uses the algorithm Paragon^TM^ for database search and Progroup^TM^ for data grouping. Data were searched using a human specific UniProt database. The false discovery rate was performed using a non-linear fitting method displaying only those results that reported a 1% global false discovery rate or better [67,68].

### 2.7. Protein Functional Interaction Network Analysis and Protein Ontology Classification

Functional interaction networks of the proteins and protein ontology classification were analyzed using the tools STRING v.10.0 database (http://string-db.org) [69] and PANTHER (http://www.pantherdb.org/), respectively. The differentially expressed proteins found for the thyroid cancers (FTC, CV-PTC, and FV-PTC) were grouped according to their molecular function, biological process, cellular component, and protein classes.

## 3. Results and Discussion

### 3.1. Deparaffinization of FFPE Tissue Samples and Protein Extraction

Although several alternatives have been developed for the deparaffinization of FFPE tissue blocks and the protein extraction [65], proteome analysis (quantitative and qualitative) continues to be a challenge. Thus, the elimination of paraffin is a crucial step in the protein extraction process from FFPE samples. The method most commonly used is the deparaffinization with xylene followed by a final rehydration step [70,71]. In the present work, the deparaffinization was carried out following the protocol reported by Mansour et al. [64], with some minor modifications. Besides not using a non-polar toxic organic solvent such as xylene, the deparaffinization with water is easier and faster because the final rehydration step is not necessary. To the best of our knowledge, similar methods have only been used for the deparaffinization of FFPE tissue samples of colorectal cancer [64] and breast and lymph nodes. Importantly, this is the first time that FFPE thyroid samples have been deparaffinized with water.

For their subsequent analysis, proteins from deparaffinized tissue samples have to be extracted. The protein extraction could be influenced by the pH value and the detergents, denaturing, and reducing agents employed. In the present work, proteins from deparaffinized tissue samples were extracted with a buffer whose composition was 20 mM Tris-HCl pH 8.8, 2% SDS, 1% CHAPS, 200 mM DTT, 200 mM glycine, and a mixture of protease inhibitors [65]. SDS was combined with reducing agents like DTT and detergents like CHAPS. In this way, some authors have suggested that buffers that include high concentrations of SDS [72] and a high pH [71,73] could induce better protein recovery.

Furthermore, the buffer composition, the temperature and the introduction of physical elements could also modulate the protein extraction process [70]. In this way, the combination of the mechanical disaggregation (TissueLyser) and the chemical action of the buffer solution improves the tissue disaggregation and facilitates the protein extraction. The long time heating the samples with buffers and the subsequent freezing of the samples overnight also develop an important role in the protein extraction process [72].

### 3.2. AuNP-, AgNP-, and FeNP-Protein Corona Identification by Mass Spectrometry Analysis

As a proof of principle to set the technique on FFPE thyroid samples, the number of samples analyzed in the present study was small. In particular, five pooled tissue extracts from normal thyroid samples (normal controls (NCs)) (*n* = 5), FTA samples (*n* = 4), FTC samples (*n* = 5), CV-PTC samples (*n* = 5), and FV-PTC samples (*n* = 3) were processed as described in Section 2.5 (Figure 2). After the ex vivo incubation of AgNPs (9.66 ± 1.77 nm), AuNPs (7.55 ± 0.70 nm), and FeNPs (8.25 ± 0.78 nm) with the reduced and alkylated proteins from the pooled tissue extracts, the PC formation took place.

**Figure 3:**
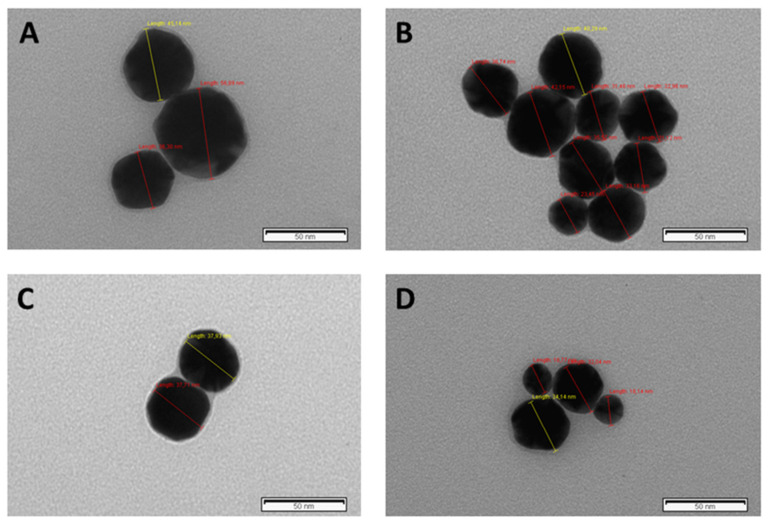
Negative stain TEM imaging of corona-coated AgNPs (9.66 ± 1.77) recovered post-incubation with pooled tissue extracts from normal thyroid tissue (**A**,**B**) and pooled tissue extracts from follicular thyroid carcinoma (FTC) samples (**C**,**D**). Red and yellow lines show the nanoparticles’ diameter.

The resultant protein corona-coated AgNPs, AuNPs, and FeNPs were centrifuged and structurally characterized by transmission electron microscopy (TEM). As a result of the PC formation, an increase in the size of AgNPs (9.66 ± 1.77 nm), AuNPs (7.55 ± 0.70 nm), and FeNPs (8.25 ± 0.78 nm) was observed (see Figures S2–S4 (Supplementary Materials)). In particular, an increase in the size of AgNPs was observed from 9.66 ± 1.77 nm to 22.03 ± 7.53 nm (normal tissue samples) and 34.81 ± 8.88 nm (FTC samples) (see Figure 3 and Figure S2 (Supplementary Materials)).

The fraction of proteins bound to the nanoparticle surfaces, named protein coronas (PCs), was separated by 1D-SDS-PAGE, digested with trypsin, and identified by LC-MS/MS analysis.

**Figure 4:**
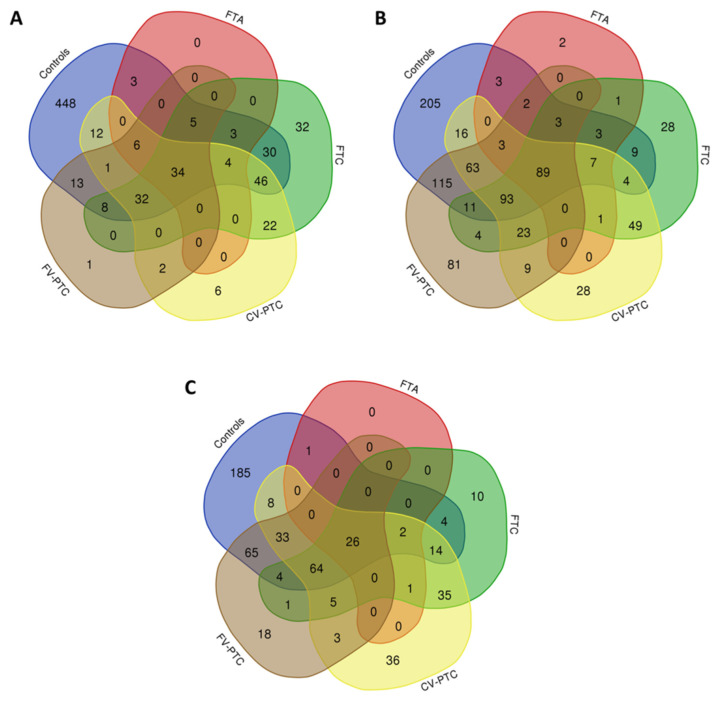
Venn diagrams showing the number of shared and specific proteins identified in the PCs formed after the interaction of AgNPs (9.66 ± 1.77) (**A**), AuNPs (7.55 ± 0.70) (**B**), and FeNPs (8.25 ± 0.78) (**C**) with pooled tissue extracts from normal thyroid samples (controls), FTA samples, and samples from different types of thyroid cancers (FTC, CV-PTC, and FV-PTC).

A total of 645, 55, 216, 165, and 102 proteins were identified on the surface of AgNPs (9.66 ± 1.77) after their incubation with pooled tissue extracts from normal controls (NCs) and FTA, FTC, CV-PTC, and FV-PTC samples, respectively (see Table 1 and Table S1 (Supplementary Materials)). From them, thirty-four were commonly found in all sample groups (see Figure 4A). Furthermore, fractionation of the proteome using AgNPs (9.66 ± 1.77 nm) resulted in the identification of 32, 6, and 1 specific proteins of FTC, CV-PTC, and FV-PTC, respectively (see Figure 4A). Similarly, fractionation of the proteome using AuNPs (7.55 ± 0.70 nm) permitted the identification of 2, 28, 28, and 81 specific proteins of FTA, FTC, CV-PTC, and FV-PTC, respectively (see Figure 4B). With FeNPs (8.25 ± 0.78 nm), ten, 36, and 18 specific proteins were identified for FTC, CV-PTC, and FV-PTC, respectively (see Figure 4C).

The combination of these results showed that a total of 2, 57, 63, and 91 potential biomarkers were found for FTA, FTC, CV-PTC, and FV-PTC, respectively (see Figure S5 (Supplementary Materials), Table 2, and Tables S1 and S2 (Supplementary Materials)). Importantly, the heterogeneous nuclear ribonucleoprotein C-like 2 (HNRNPCL2) was commonly identified for FTA and CV-PTC and the 3-ketoacyl-CoA thiolase, mitochondrial (ACAA2), for FTC and FV-PTC. Furthermore, seven biomarkers were commonly identified for FTC and CV-PTC named acyl-coenzyme A thioesterase 13 (ACOT13), ATP synthase subunit e, mitochondrial (ATP5ME), cytochrome b-c1 complex subunit 8 (UQCRQ), heat shock protein 75 kDa, mitochondrial (TRAP1), histidine triad nucleotide-binding protein 2, mitochondrial (HINT2), peptidyl-prolyl cis-trans isomerase F, mitochondrial (PPIF), and protein ABHD11 (ABHD11).

Only two potential biomarkers of FTA were identified in the PC of AuNPs: the aldo-keto reductase family 1 member C1 (AKR1C1) and the heterogeneous nuclear ribonucleoprotein C-like 2 (HNRNPCL2) (see Table 2). In particular, AKR1C1 develops an important role in the maintenance of the steroid hormone homeostasis, prostaglandin metabolism, and the metabolic activation of polycyclic aromatic hydrocarbons [74,75].

### 3.3. Functional Annotation and Gene Ontology Analysis

**Figure 5:**
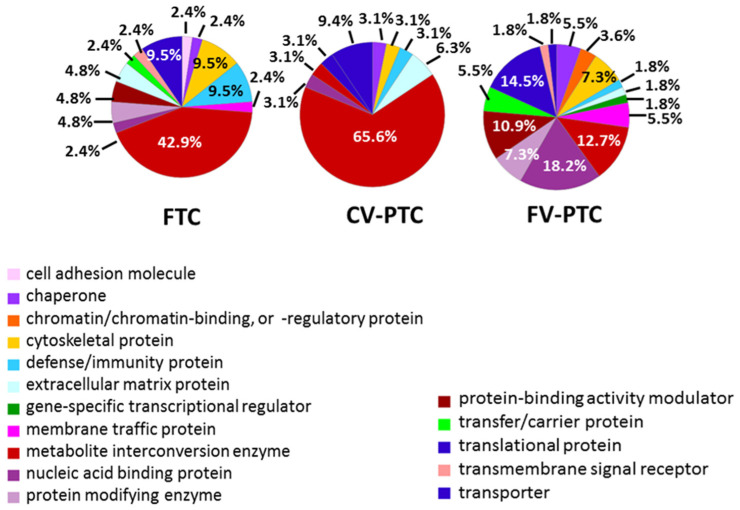
Classification according to the protein class of the proteins (biomarkers) identified in samples of FTA and samples from different types of thyroid cancers (FTC, CV-PTC, and FV-PTC).

**Figure 6:**
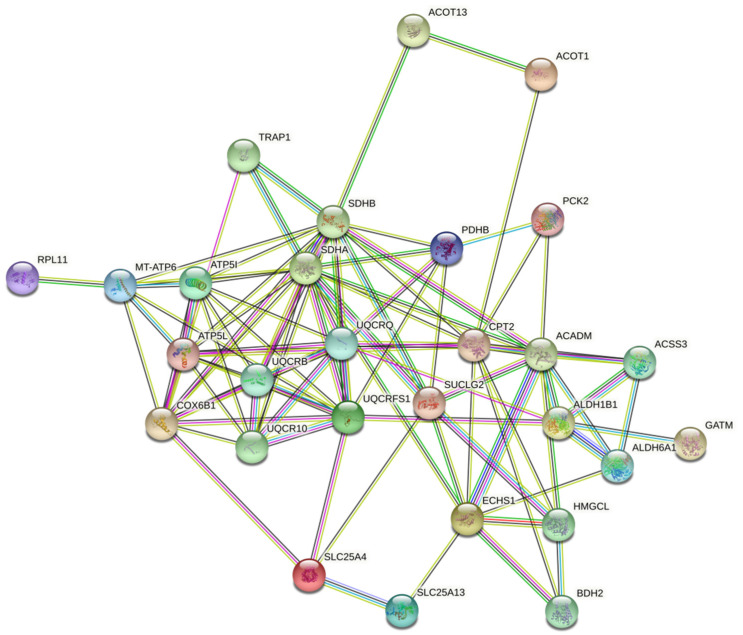
The cluster of proteins implicated in the cancer metabolic reprogramming found in the protein-protein interaction (PPI) network map of the genes encoding differentially regulated proteins for the CV-PTC subtype identified after the proteomic analysis of the ex vivo PCs.

GO analysis based on the molecular functions of specific proteins for FTC, CV-PTC, and FV-PTC is shown in Figure S6. The molecular functions most represented in the PCs for all subtypes were binding (45.6–26.7%) and catalytic (55.6–35.3%) activities, with the remaining categories (translational regulator, transducer, structural, and transporter) being in the minority. In all cases, protein biomarkers were concentrated in the cell (mainly in organelles) (Figure S7 (Supplementary Materials)), participating in the cellular metabolic processes and biological regulation (Figure S8 (Supplementary Materials)). As Figure 5 shows, the potential biomarkers for FTC and CV-PTC, and to a lesser extent for FV-PTC, were metabolite interconversion enzymes. Figure 6 shows the cluster of proteins implicated in the cancer metabolic reprogramming found in the protein-protein interaction (PPI) network map of the genes encoding differentially regulated proteins for the CV-PTC subtype using the STRING v.10.0 database. Clusters that contain terms and pathways related to metabolic reorganization found after the PPI network analysis of the FTC and FV-PTC subtypes are also shown in Figure S9 (Supplementary Materials).

### 3.4. Thyroid Cancer Metabolism: Proteins Implicated in the Cancer Metabolic Reprogramming

The synthesis of the thyroid hormone is an oxidative process that demands a high quantity of energy [76]. The majority of the energy required to preserve cell functions is produced within the inner mitochondrial membrane by an oxidative phosphorylation (OxPhos) process [77]. As is shown in Table 2, different proteins that are implicated in the oxidative phosphorylation were identified as the acyl carrier protein, mitochondrial (NDUFAB1) for FTC, ATP synthase subunit a (MT-ATP6), ATP synthase subunit g, mitochondrial (ATP5MG), succinate dehydrogenase (ubiquinone) flavoprotein subunit, mitochondrial (SDHA), succinate dehydrogenase (ubiquinone) iron-sulfur subunit, mitochondrial (SDHB) for CV-PTC, and ATP synthase subunit e, mitochondrial (ATP5ME) for FTC and CV-PTC.

Under physiological conditions, two processes are responsible for the existence of high levels of reactive oxygen species (ROSs) in the thyroid, on the one hand the OxPhos process, and on the other, hormonogenesis [78]. This explains the presence of several endogenous antioxidant systems in the thyroid gland, whose deficiencies produce the oxidative damage observed in thyroid tumors [79]. While in well-differentiated thyroid cancer (TC), an augmented activity of the enzymes implicated in the antioxidant defense takes place, their inactivation is characteristic of poorly differentiated tumors [80]. In the present work, some enzymes were identified as being implicated in the antioxidant defense such as the regulatory antioxidant enzyme NADPH: adrenodoxin oxidoreductase, mitochondrial (FDXR) in FTC, glutathione S-transferase kappa 1 (GSTK1) and superoxide dismutase (Mn), mitochondrial (SOD2) in CV-PTC, and the thioredoxin domain-containing protein 5 (TXNDC5) in FV-PTC.

Frequently, in high proliferating cancer cells, an increase in the consumption of glucose with the subsequent production of lactate instead of oxygen takes place, named glycolysis. This implies the suppression of the mitochondrial OxPhos. The metabolic change from the OxPhos to the glycolysis process promotes the growth and survival of cancer cells, under hypoxic and nutrient-depleted conditions. This implies the improvement in the biosynthetic fluxes and the antioxidant defense throughout fast proliferation; this is known as the “Warburg effect” [81].

In particular, hypoxia-inducible factor 1 alpha (HIF-1α) is a transcription factor implicated in this metabolic reprogramming, which develops a crucial role in the glycolysis activation and OxPhos inhibition [82,83]. While HIF-1α expression in normal thyroid tissue is not observed, it is commonly overexpressed in thyroid cancers as ATC, which are more aggressive [84]. Furthermore, the association of the HIF-1α overexpression with the distant metastasis in PTC has been described [85,86]. In the present work, a similar transcription factor named hypoxia upregulated protein 1 (HYOU1) was identified in FV-PTC. HIF-1α also activates the expression of glycolytic enzymes such as phosphoglycerate kinase (PGK), hexokinase II (HKII), lactate dehydrogenase A (LDH-A), and glucose-6-phosphate dehydrogenase (G6PDH), or glucose and lactate transporters, such as glucose transporter 1 (GLUT1) and the monocarboxylate transporter 4 (MCT4), which are overexpressed in TC [87,88,89,90]. Overall, these results show that some cells within thyroid tumors exhibit the “Warburg” phenotype, producing their energy through an aerobic glycolysis process. In the present work, the glycolytic enzymes (enzymes implicated in glycolysis) hexokinase HKDC1 (HKDC1) and pyruvate kinase (PKLR) were identified in FTC.

It was also described how the interruption of cytochrome c oxidase function prompts the “Warburg effect” and the metabolic reprogramming. Thus, a defect in the cytochrome c oxidase (CcO) complex could promote tumor progression [91]. In relation to this metabolic process, cytochrome b-c1 complex subunit 8 (UQCRQ), cytochrome c oxidase subunit NDUFA4 (NDUFA4), and cytochrome c oxidase subunit 7A1, mitochondrial (COX7A1), have been identified in FTCs. While cytochrome b-c1 complex subunit Rieske, mitochondrial (UQCRFS1), cytochrome b-c1 complex subunit 9 (UQCR10), cytochrome b-c1 complex subunit 7 (UQCRB), cytochrome b-c1 complex subunit 8 (UQCRQ), and cytochrome c oxidase subunit 6B1 (COX6B1) have been identified in CV-PTC, NADH-cytochrome b5 reductase 1 (CYB5R1) has been found in FV-PTC.

For tumor growth, tumor cells need a high fatty acid turnover rate to obtain all the energetic and synthetic requirements [92]. Although the patients’ lipid profile is associated with various types of carcinoma [93], its utility as a diagnostic tool for thyroid carcinoma is unknown [94].

In the present work, several proteins associated with lipid metabolism discriminated amongst different types of thyroid lesions. These proteins were D-beta-hydroxybutyrate dehydrogenase, mitochondrial (BDH1), fatty acid-binding protein, heart (FABP3), propionyl-CoA carboxylase alpha chain, mitochondrial (PCCA), propionyl-CoA carboxylase beta subunit (PCCB), and succinyl-CoA:3-ketoacid coenzyme A transferase 1, mitochondrial (OXCT1), for FCT. For CV-PTC, the proteins were acyl-CoA synthetase short-chain family member 3, mitochondrial (ACSS3), acyl-coenzyme A thioesterase 1 (ACOT1), carnitine O-palmitoyltransferase 2, mitochondrial (CPT2), enoyl-CoA hydratase, mitochondrial (ECHS1), estradiol 17-beta-dehydrogenase 8 (HSD17B8), 3-hydroxybutyrate dehydrogenase type 2 (BDH2), hydroxymethylglutaryl-CoA lyase, mitochondrial (HMGCL), and medium-chain specific acyl-CoA dehydrogenase, mitochondrial (ACADM). The protein acyl-coenzyme A thioesterase 13 (ACOT13) was associated with both FTC and CV-PTC. The protein 3-ketoacyl-CoA thiolase, mitochondrial (ACAA2), was associated with FTC and FV-PTC.

Importantly, all these tumor-related proteins we identified could participate in the control of cancer metabolic reprogramming, the determination of clinical phenotypes of thyroid cancer, and the level of its aggressiveness [95].

### 3.5. Proteins Implicated in Thyroid Cancer Progression and Metastasis

In the present work, different proteins related to tumor progression were identified. For example, thrombospondin-1 (THBS1), periostin (POSTN), ferritin heavy chain (FTH1), and ferritin light chain (FTL) were found in FTC patients.

THBS1, in particular, was found to be closely associated with the focal adhesion pathway, responsible for the tumor cell invasion and metastasis [96]. POSTN also develops an important role in angiogenesis, invasiveness, tumor growth, and metastasis [97]; high expression of POST was related to increased tumor aggressiveness such as extra thyroid invasion, lymph node metastasis, and higher grade staging in thyroid carcinoma [98]. Furthermore, although serum ferritin may not be a tumor marker for thyroid cancer, serum ferritin levels seem to be sensitive to the presence of metastasis and histological diagnosis. Importantly, the classification of thyroid cancer patients into different histological types showed higher ferritin levels in FTC when compared to PTC [99].

In the present work, one protein of the 14-3-3 family, the 14-3-3 protein eta (YWHAH), was identified in the CV-PTC subtype. YWHAH was found to be overexpressed in multiple types of cancers, developing an important role in the regulation of different biological pathways implicated in cancer progression [100] and was also related to a poor prognosis in cancer patients [101].

In the FV-PTC subtype, the proteins that were involved in apoptotic machinery, tumor progression, cell transformation, and invasion were: programmed cell death 4 (PDCD4), programmed cell death 6 (PDCD6), and CD44 antigen (CD44). While one study found that the PDCD4 protein was downregulated in PTC [102], other studies showed superior expression of the cell adhesion molecule CD44 in PTC [103,104,105].

Several studies have shown a role for blood coagulation proteins in tumor progression [106]. In the present work, the blood coagulation-related proteins identified were antithrombin-III (SERPINC1) for FTC, vitamin K-dependent protein S (PROS1) for CV-PTC, and serpin B6 (SERPINB6) and serpin H1 (SERPINH1) for FV-PTC. While antithrombin was found to be a modulator of tumor cell migration and invasion [107], PROS1 was highlighted as a potential biomarker for thyroid nodules malignancy [108] and thyroid cancer [109].

## 4. Conclusions

This study showed that FFPE thyroid tissue samples contain a large amount of hidden information. Characterization and identification of protein molecules that interact with AgNPs (9.66 ± 1.77), AuNPs (7.55 ± 0.70), and FeNPs (8.25 ± 0.78) from tissue extracts of normal thyroid tissue and FTA, FTC, CV-PTC, and FV-PTC samples provide mechanistic insights into the biology of cancer metabolic reprogramming, tumor growth, and metastasis. Importantly, proteins related to these biological pathways could be potential molecular targets of different thyroid malignancies. These proteins could potentially be targeted for maximal therapeutic benefit in thyroid cancer treatment. However, due to the small number of tissues analyzed, the potential markers identified should be investigated in larger series. After their validation, these proteins could be also investigated in cytological samples to identify reliable markers for pre-surgical diagnosis in indeterminate thyroid nodules.

## Figures and Tables

**Table 1:** The number of proteins (total and specific) identified in the protein patterns of the ex vivo formed coronas of AgNPs (9.66 ± 1.77), AuNPs (7.55 ± 0.70), and FeNPs (8.25 ± 0.78) after their incubation with the pooled tissue extracts from normal thyroid samples (controls), samples from FTA, and samples from different types of thyroid cancers: FTC, CV-PTC, and FV-PTC.

	NPs	AgNPs		AuNPs		FeNPs	
Groups		Total	Specific	Total	Specific	Total	Specific
Controls		645	448	626	205	406	185
FTA		55	0	114	2	30	0
FTC		216	32	325	28	166	10
CV-PTC		165	6	385	28	227	36
FV-PTC		102	1	496	81	219	18

**Table 2:** Specific molecular targets identified for the different thyroid malignancies (FTA, FTC, CV-PTC, and FV-PTC). Proteins in bold were identified in more than one subtype.

FTA	CV-PTC	FV-PTC
AuNPs (*n* = 2)	AgNPs-FeNPs (*n* = 3)	AgNPs-AuNPs-FeNPs (*n* = 1)
AKR1C1, **HNRNPCL2**	ACSS3, GATM, ACADM	IGSF1
FTC	AuNPs-FeNPs (*n* = 4)	AuNPs-FeNPs (*n* = 7)
AgNPs-AuNPs-FeNPs (*n* = 4)	CISD1, UQCRFS1, **HINT2**, VDAC3	DBI, APOA2, DPYSL3, RPL3,
FABP3, FTH1, FTL, HKDC1		HNRNPUL2, PCSK2, PSMA5
AgNPs-AuNPs (*n* = 2)	AgNPs (*n* = 3)	AuNPs (*n* = 73)
DIABLO, SFXN1	SLC25A4, **HNRNPCL2**, KRT15	ADK, ARL8B, AHSG, NARS1, DARS1, DDX1, DDX39A, EPRS1, PRKAR2A, CD44, CLTA, COL14A1, MACROH2A2, CST3, ACO1, ARMT1, DDB1, DUOX2, DNM2, EIF2AK4, ECM1, CAPZB, FUBP3, FBP1, GPD2, GRHPR, GNA13, HSPA4, H2BU1, ITIH1, SSB, CYB5R1, HMGN1, PSIP1, DDX5, PDCD4, PDCD6, PSMA3, PSMA7, SEC31A, PSMD2, PSMD6, PSMD11, RPL26L1, RPL38, SRRM1, PPP1CB, SERPINB6, SERPINH1, U2AF2, SND1, Q99832, CCT3, CCT8, TXNDC5, TMSB4X, TWF1, UGP2, RAD23B, VARS1, GC, VWA1, ATP6V1A, PSMD12, ARHGDIA, PLS1, FLOT1, PSMA6, WARS1, LRRC47, EFHD2, RNPEP, DDAH1
AgNPs-FeNPs (*n* = 2)	AuNPs (*n* = 24)	
H1-3, POSTN	**ACOT13**, AHCYL2, ATPAF2, CPT2, UQCR10, ALDH18A1, DNPH1, DSTN, HSD17B8, FBN2, LGALS1, GSTK1, GRPEL1, HDHD3, BDH2, HMGCL, RNASE10, NADK2, PLGRKT, FMC1, NIPSNAP3A, RPL11, SDHB, PROS1	
AuNPs-FeNPs (*n* = 1)		
ALDH4A1		
AgNPs (*n* = 24)		
**ACOT13**, SERPINC1 GOT1, **ATP5ME**, **UQCRQ**, NDUFA4 BDH1, ETFA, FH, **TRAP1**, **HINT2** IDH3A, **ACAA2**, MTCH2, SLC25A11, MYL6B, NPC2, **PPIF** PCCA, **ABHD11**, PKLR, ALDH5A1 OXCT1, TUBB2B		
	FeNPs (*n* = 29)	
	ACOT1, SLC25A31, ALDH1B1, MT-ATP6, ATP5ME, **ATP5MG**, SLC25A13, C1QBP, CKB, UQCRB, **UQCRQ**, COX6B1, ECHS1, **TRAP1**, H2BC15, IGHA2, ALDH6A1, IMMT, ABHD10, **PPIF**, PCK2, **ABHD11**, YWHAH, PDHB, SUCLG2, SDHA, SOD2, YKT6, TFAM	
AuNPs (*n* = 21)		
ACTC1, NDUFAB1, CASP14, CNTNAP4, CKMT2, COX7A1, DUT GLUD2, GNB4, IGKV2D-28 IGKV3-11, KRT77, LAMP2, CD14 FDXR, PCCB, FAM162A, XP32 STOML2, THBS1, TPM2		
FeNPs (*n* = 3)		
IGKV3-15, IGKV3-20, TPP1		FeNPs (*n* = 10)
		ARF5, RPLP0P6, EIF5A2, H2AX, HYOU1, ILF3, **ACAA2**, SET, RPL12, WDR1

## Supplementary Materials

The following are available online at https://www.mdpi.com/2079-4991/10/12/2370/s1: Figure S1. Flowchart depicting the separation by 1D-SDS-PAGE and digestion with trypsin of the corona proteins associated with AgNPs (9.66 ± 1.77), AuNPs (7.55 ± 0.70), and FeNPs (8.25 ± 0.78) prior to the mass spectrometry (LC-MS/MS) identification, Figure S2. Characterization data and TEM images of (a) bare AgNPs, (b) Ag@PC controls, AgNPs after their incubation with pooled tissue extracts of disease-free individuals (controls), and (c) Ag@PC patients, pooled tissues extracts of patients (diagnosed with FCT) in the aqueous phase, Figure S3. Characterization data and TEM images of (a) bare AuNPs, (b) Au@PC controls, AgNPs after their incubation with pooled tissue extracts of disease-free individuals (controls), and (c) Au@PC patients, pooled tissues extracts of patients (diagnosed with FCT) in the aqueous phase, Figure S4. Characterization data and TEM images of (a) bare FeNPs, (b) Fe@PC controls, AgNPs after their incubation with pooled tissue extracts of disease-free individuals (controls), and (c) Fe@PC patients, pooled tissues extracts of patients (diagnosed with FCT) in the aqueous phase, Figure S5. Venn diagrams representing the number of shared and specific molecular targets identified for the different tumor thyroid malignancies (FTA, FTC, CV-PTC, and FV-PTC), Figure S6. Classification according to the molecular function of the potential molecular targets identified in patients with FTA and patients with different types of thyroid cancers (FTC, CV-PTC, and FV-PTC), Figure S7. Classification according to the cellular component of the potential molecular targets in patients with FTA and patients with different types of thyroid cancers (FTC, CV-PTC, and FV-PTC), Figure S8. Classification according to the biological process of the potential molecular targets identified in patients with FTA and patients with different types of thyroid cancers (FTC, CV-PTC, and FV-PTC), Figure S9. Significant clusters that contain terms and pathways related to the metabolic reorganization in the FTC (A), CV-PTC (B), and FV-PTC (C) PPI network analysis using the STRING v.10.0 database, Table S1. Shared and specific molecular targets identified for the different tumor thyroid malignancies (FTA, FTC, CV-PTC, and FV-PTC), Table S2. Specific molecular targets identified for the different thyroid malignancies (FTA, FTC, CV-PTC, and FV-PTC). Proteins in bold were identified in more than one subtype.
