# Supplementary material for: A Novel Nanoproteomic Approach for the Identification of Molecular Targets Associated with Thyroid Tumors
Source: Nanomaterials (Basel). 2020 Nov 28;10(12):2370. doi: 10.3390/nano10122370 (PMC7761166; doi:10.3390/nano10122370)
Supplement: Supplementary file 1 [file nanomaterials-10-02370-s001.pdf]

Supplementary Materials: A Novel Nanoproteomic Approach for the Identification of Molecular Targets Associated with Thyroid Tumors

María García-Vence, María del Pilar Chantada-Vázquez, José Manuel Cameselle-Teijeiro, Susana B. Bravo and Cristina Núñez

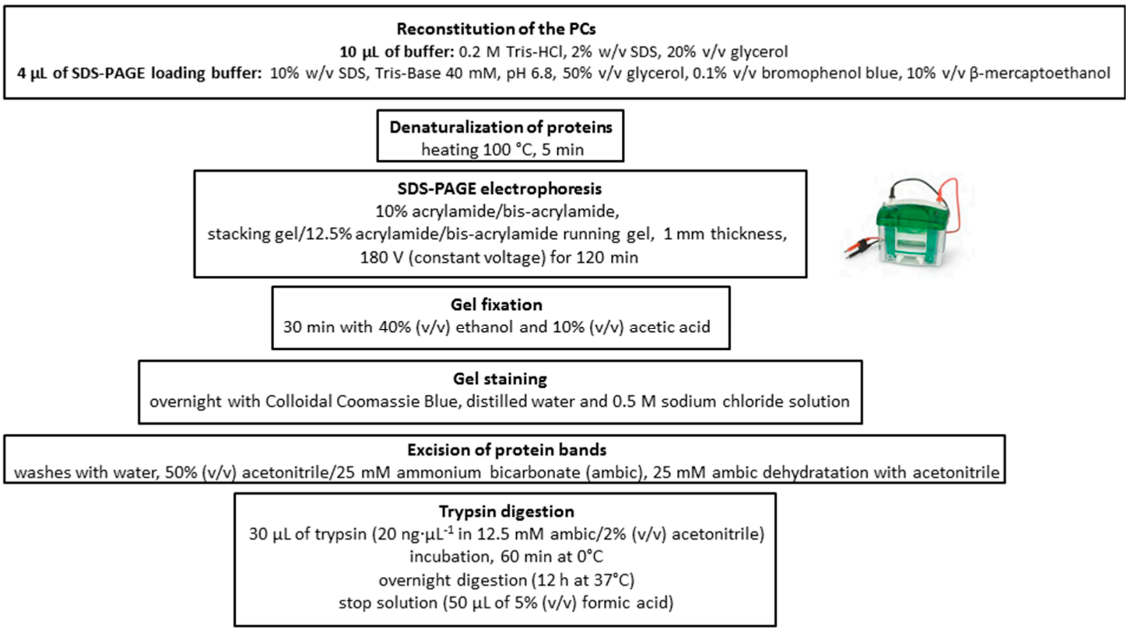

Figure S1. Flowchart depicting the separation by 1D-SDS-PAGE and digestion with trypsin of the corona proteins associated with AgNPs (9.66 ± 1.77), AuNPs (7.55 ± 0.70) and FeNPs (8.25 ± 0.78) previous to the mass spectrometry (LC-MS/MS) identification.

Figure S2. Characterization data and TEM images of a) bare **AgNPs**, b) **Ag@PC controls**: AgNPs after their incubation with pooled tissue extracts of disease-free individuals (controls), and c) **Ag@PC patients**: pooled tissues extracts of patients (diagnosed with FCT) in aqueous phase.

.	**AgNPs**	**Ag@PC controls**	**Ag@PC patients**
1	10.99	26.01	26,71
2	8.19	23.10	35,88
3	11.83	35.33	44,07
4	8.55	26.36	47,10
5	11.83	36.78	33,77
6	11.64	25.76	36,97
7	6.91	32.90	49,56
8	8.19	29.19	35,18
9	8.17	22.23	49,45
10	8.21	15.64	38,06
11	9.30	26.51	20,52
12	8.40	28.52	21,39
13	13.76	27.49	25,36
14	11.47	10.94	30,76
15	7.89	23.83	30,49
16	9.58	27.06	26,01
17	11.27	17.93	46,56
18	9.86	27.71	36,27
19	9.02	35.07	26,02
20	10.61	19.18	45,23
21	13.02	14.14	25,39
22	7.94	18.77	49,31
23	11.17	12.04	32,57
24	9.45	18.14	38,56
25	9.76	27.34	43,42
26	7.50	25.58	28,25
27	12.10	11.35	44,86
28	8.48	10.36	24,53
29	10.48	20.60	47,77
30	10.25	18.30	31,31
31	8.64	15.23	27,19
32	13.49	11.63	34,19
33	10.39	22.96	32,23
34	9.16	20.16	23,95
35	7.89	15.14	38,01
36	8.40	14.96	37,83
37	7.66	11.59	45,14
38	7.18	25.69	20,69
39	8.75	14.98	36,30
40	9.07	34.95	25,63
Count	40	40	40
Mean	9.66	22.03	34.81
Minimum	6.91	10.36	20.52
Maximum	13.76	36.78	49.45
Standard Deviation	1.77	7.53	8.88

a) TEM image of bare **AgNPs** in aqueous phase.

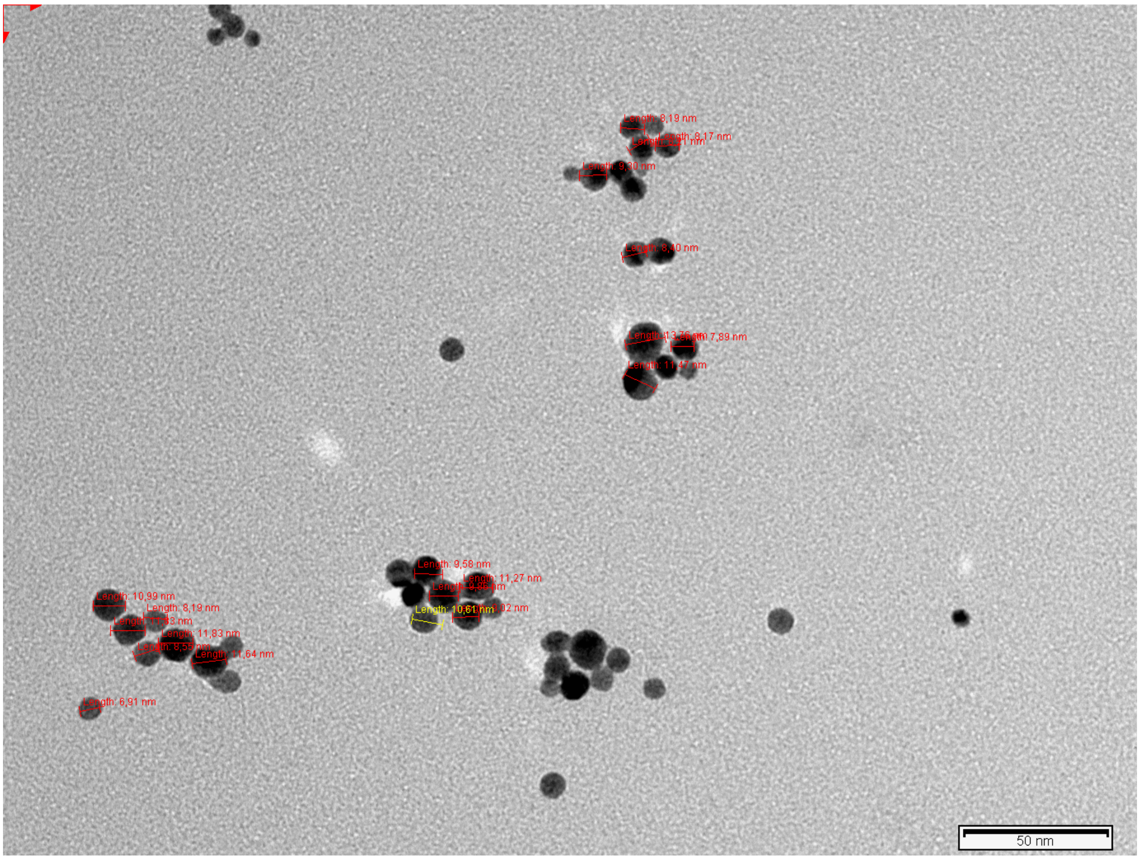

b) TEM images of AgNPs after their incubation with pooled tissue extracts of disease-free individuals (controls) in aqueous phase: AgNPs-controls.

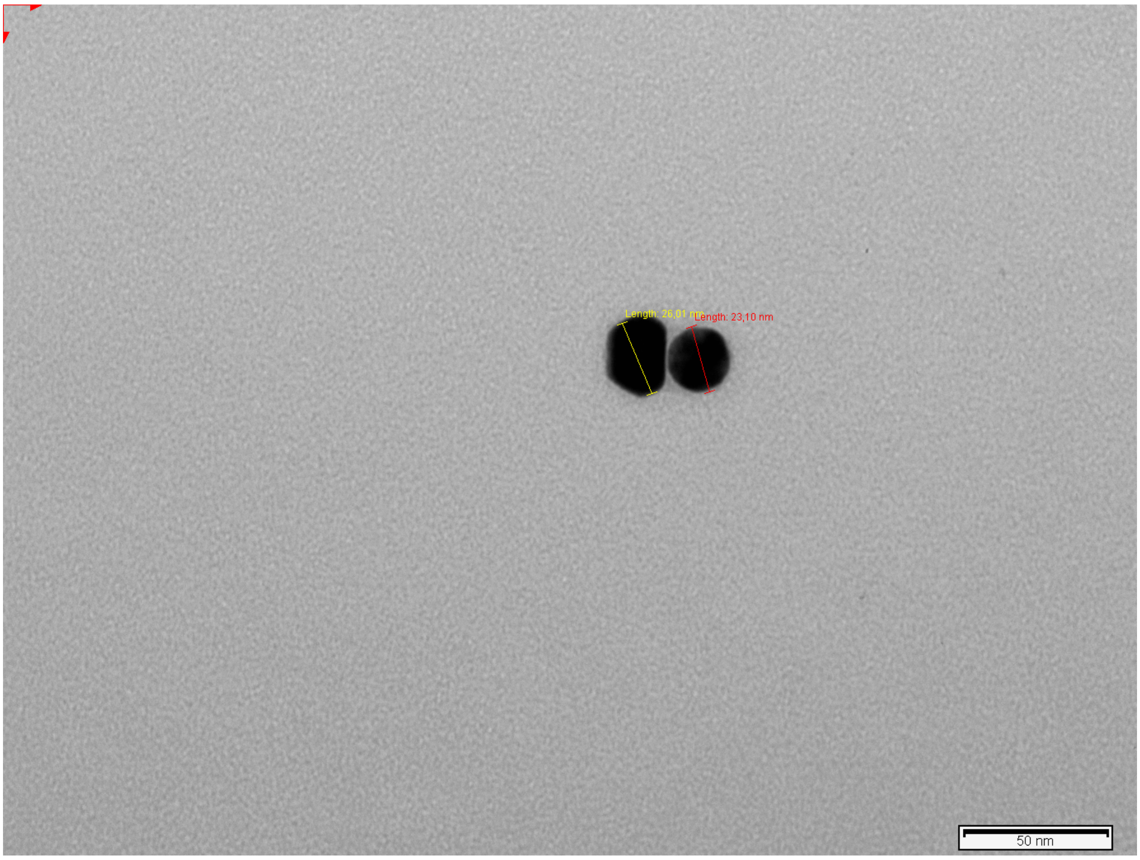

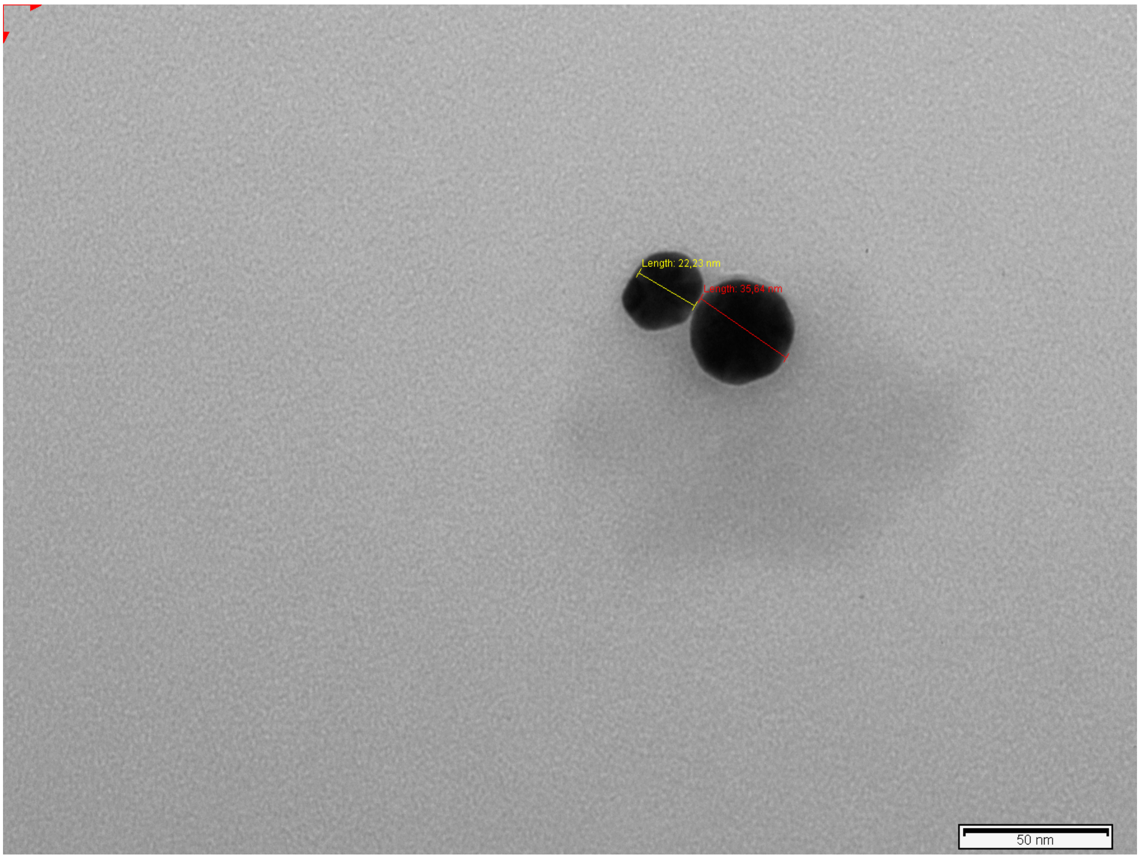

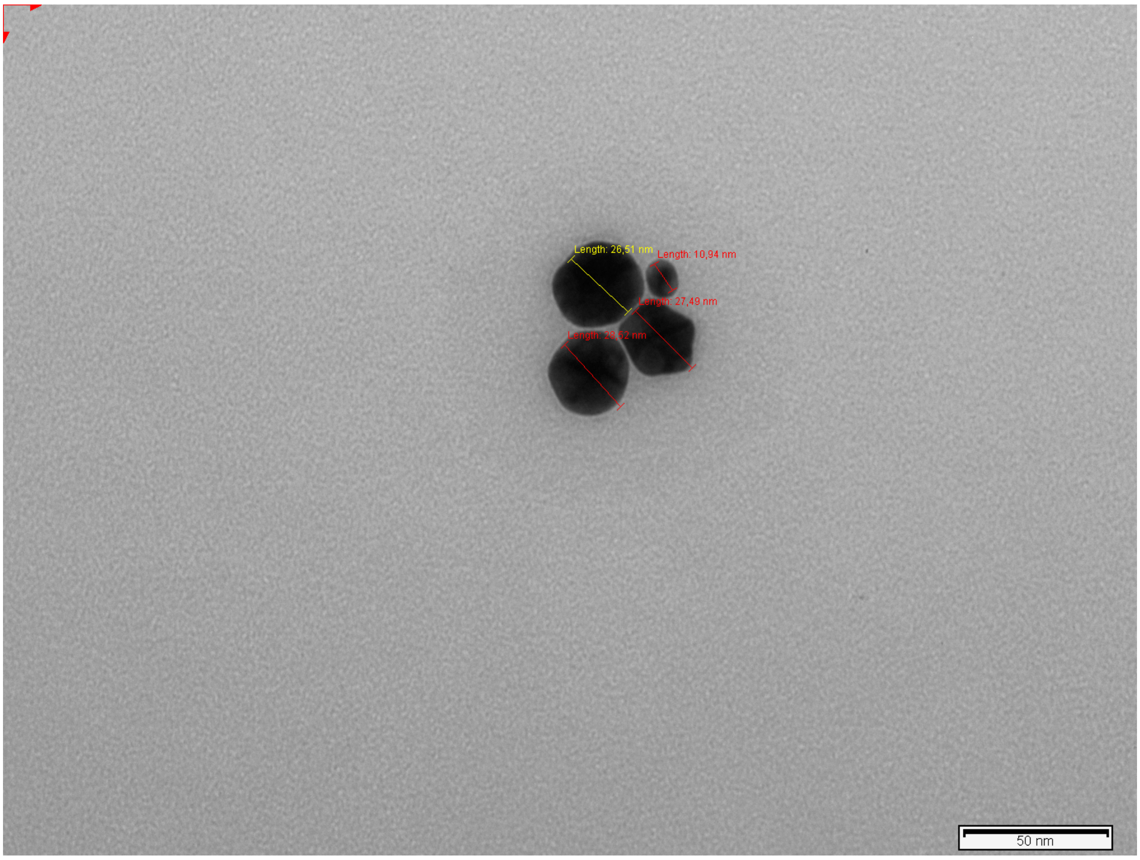
Length: 26,51 nm
Length: 10,94 nm
Length: 27,49 nm
Length: 28,52 nm
50 nm

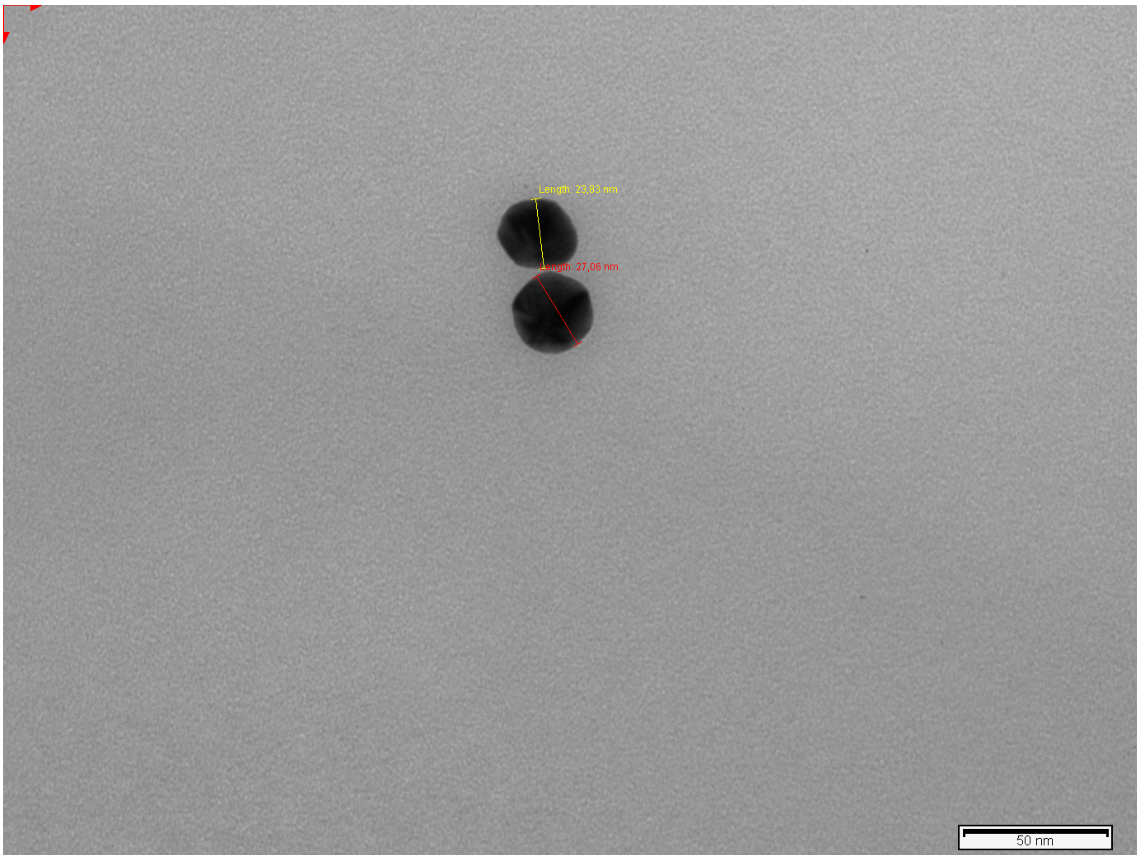
Length: 23,83 nm
Length: 27,06 nm
50 nm

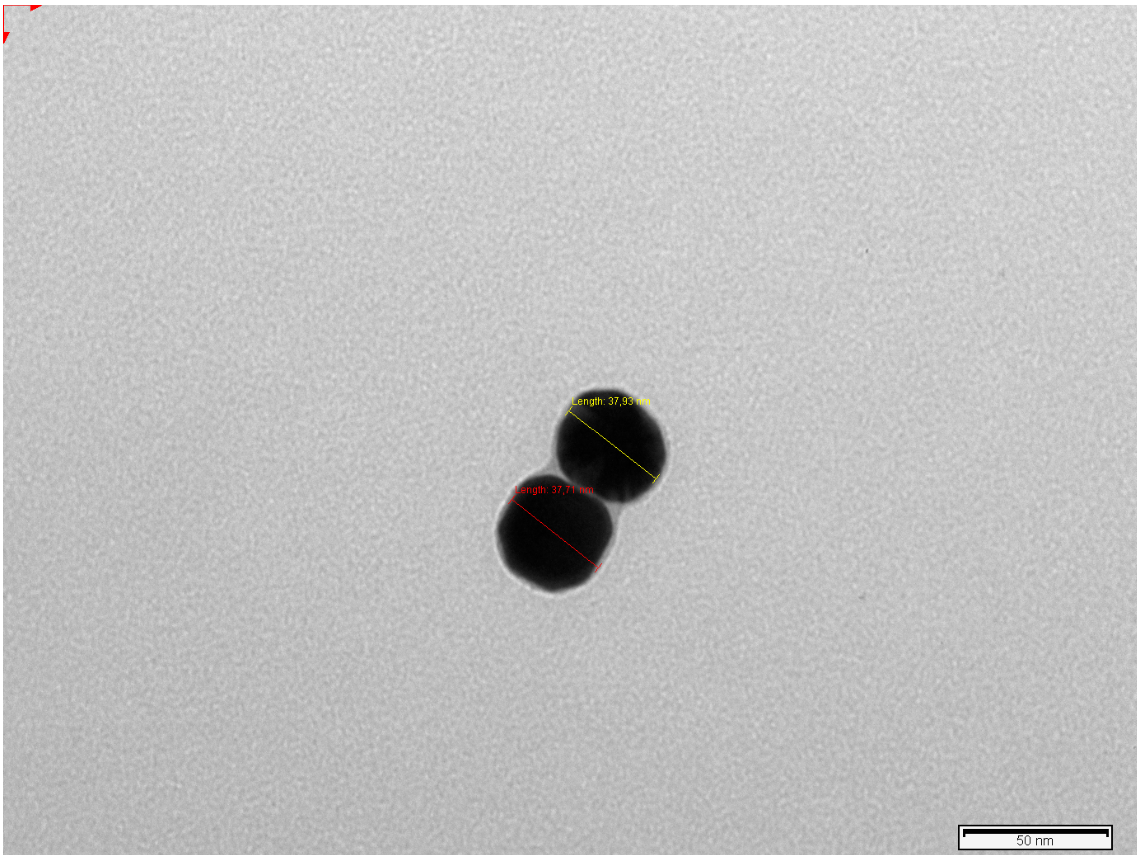
Length: 37,93 nm
Length: 37,71 nm
50 nm

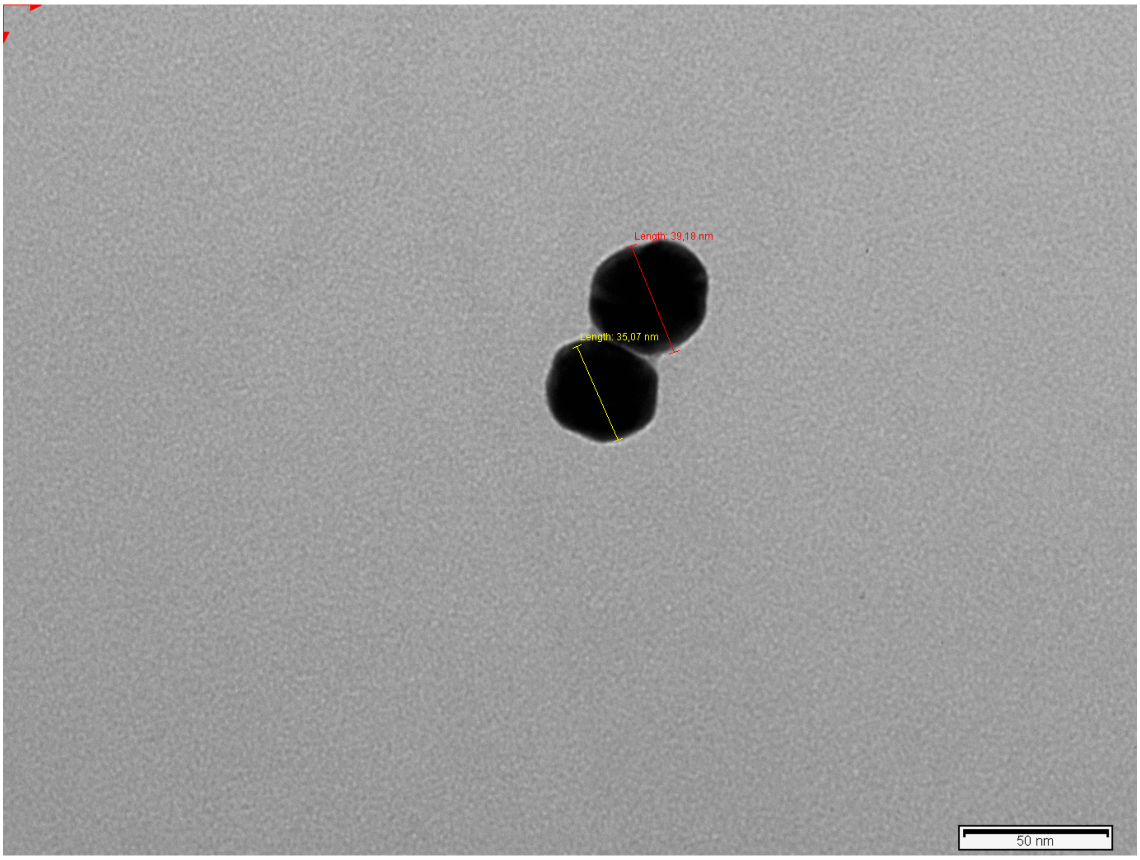
Length: 39,18 nm
Length: 35,07 nm
50 nm

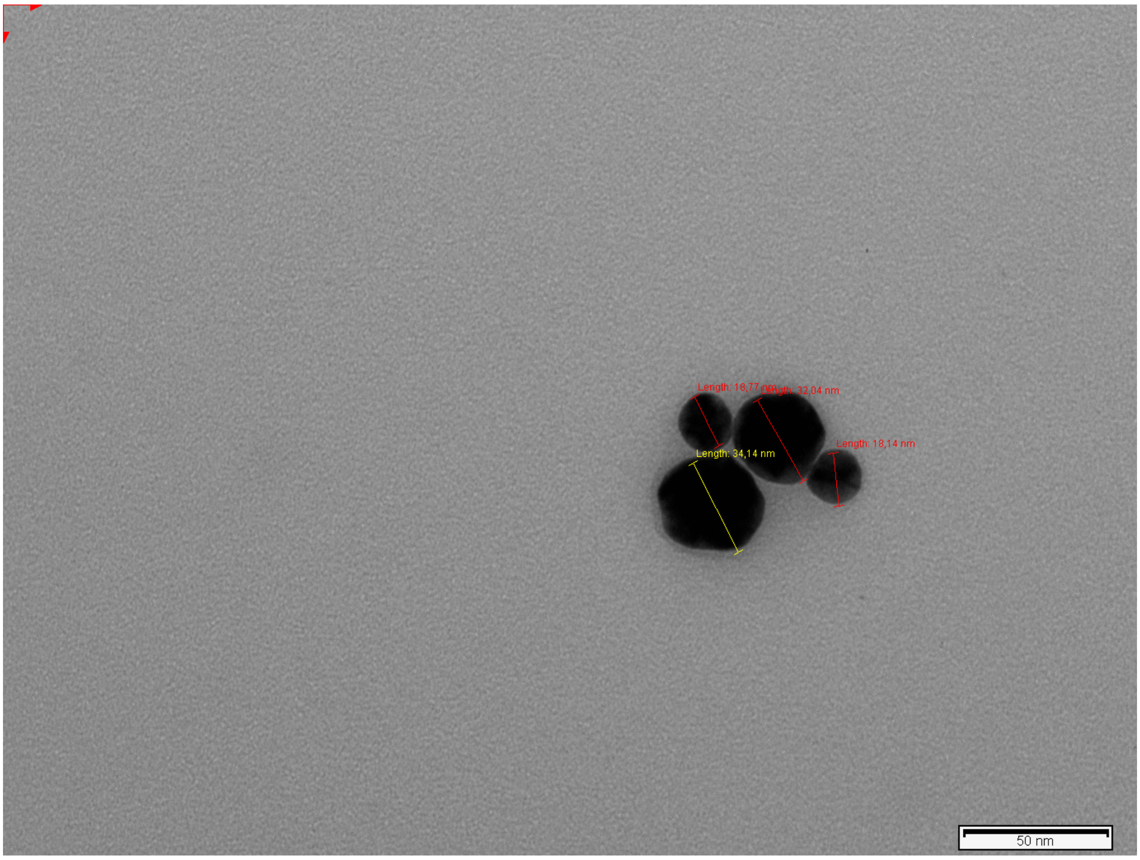
Length: 18,77 nm
Length: 32,04 nm
Length: 18,14 nm
Length: 34,14 nm
50 nm

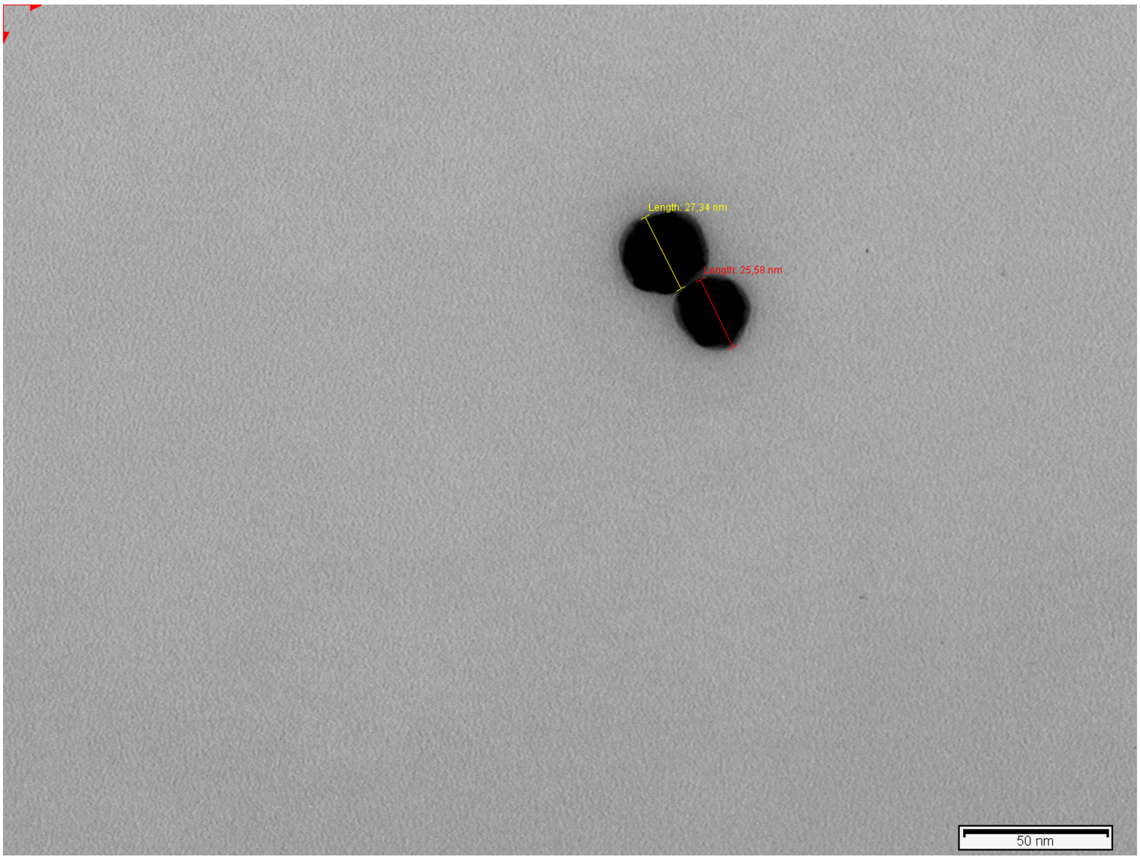
Length: 27,34 nm
Length: 25,58 nm
50 nm

c) TEM images of AgNPs after their incubation with pooled tissue extracts of patients (diagnosed with FCT) in aqueous phase: AgNPs-patients.

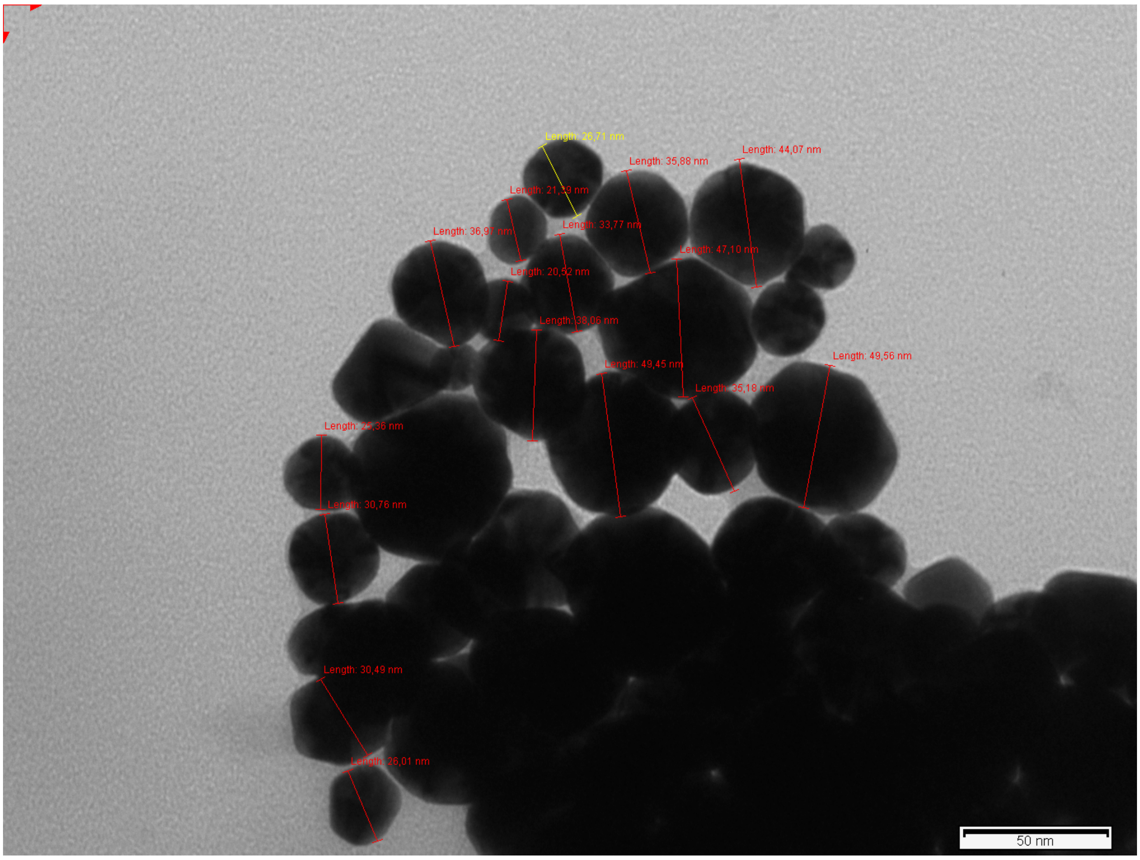

Figure S3. Characterization data and TEM images of a) bare **AuNPs**, b) **Au@PC controls**: AgNPs after their incubation with pooled tissue extracts of disease-free individuals (controls), and c) **Au@PC patients**: pooled tissues extracts of patients (diagnosed with FCT) in aqueous phase.

	**AuNPs**	**Au@PC controls**	**Au@PC patients**
1	7.35	10.86	9.49
2	7.04	8.66	11.50
3	6.33	9.19	10.03
4	6.30	8.37	8.41
5	7.13	7.42	6.95
6	7.13	8.99	9.41
7	7.41	7.80	8.87
8	8.01	8.78	8.57
9	7.74	6.53	9.84
10	8.06	7.94	8.66
11	6.33	7.52	8.82
12	6.70	8.38	7.53
13	7.85	9.88	8.38
14	7.66	9.62	8.90
15	8.07	8.87	10.68
16	8.01	8.60	8.90
17	7.83	9.49	9.44
18	6.58	8.17	9.82
19	7.35	8.78	9.55
20	8.64	7.73	11.48
21	6.58	9.35	9.43
22	8.59	7.89	8.89
23	7.81	8.83	10.13
24	8.01	8.95	9.75
25	8.52	9.95	8.69
26	7.00	10.01	8.26
27	7.98	9.98	10.89
28	6.98	9.95	9.75
29	8.19	8.75	9.64
30	8.68	7.89	8.98
31	6.81	10.11	9.15
32	7.39	9.73	10.06
33	6.82	10.98	8.96
34	7.44	9.01	8.88
35	8.19	8.98	9.23
36	8.58	8.63	9.56
37	7.39	9.63	8.76
38	8.84	8.22	8.36
39	7.17	10.15	10.02
40	7.59	9.86	9.35
Count	40	40	40
Mean	7.55	8.96	9.29
Minimum	6.30	7.42	7.53
Maximum	8.84	10.86	11.50
Standard Deviation	0.70	0.97	0.91

a) bare AuNPs in aqueous phase.

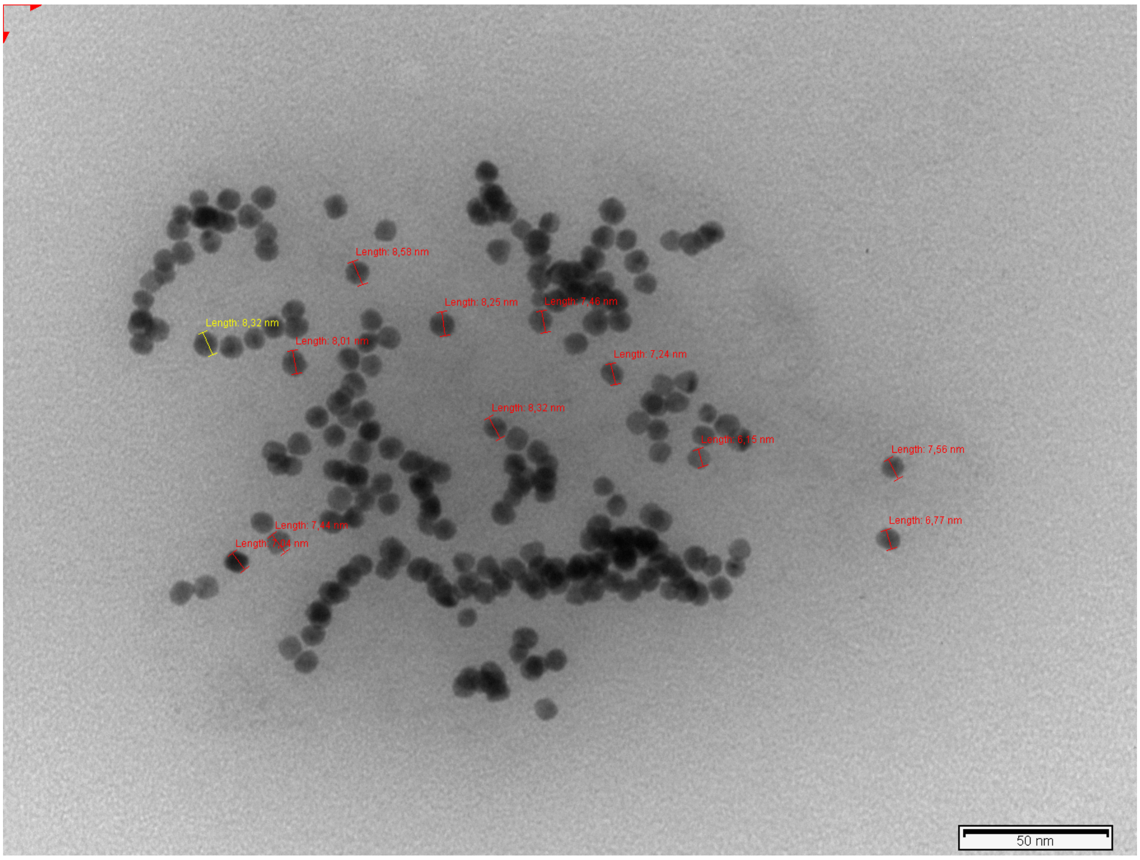

b) TEM images of AuNPs after their incubation with pooled tissue extracts of disease-free individuals (controls) in aqueous phase: AuNPs-controls.

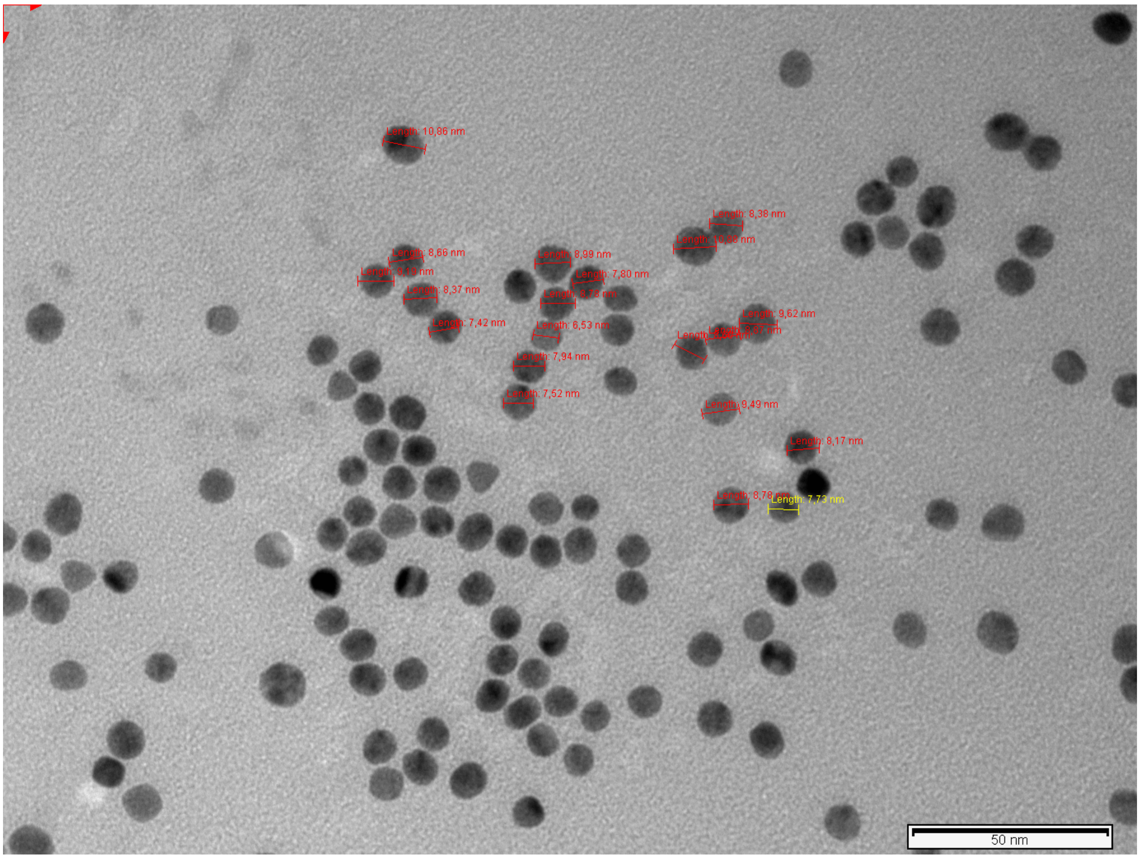

c) TEM images of AuNPs after their incubation with pooled tissue extracts of patients (diagnosed with FCT) in aqueous phase: AuNPs-patients.

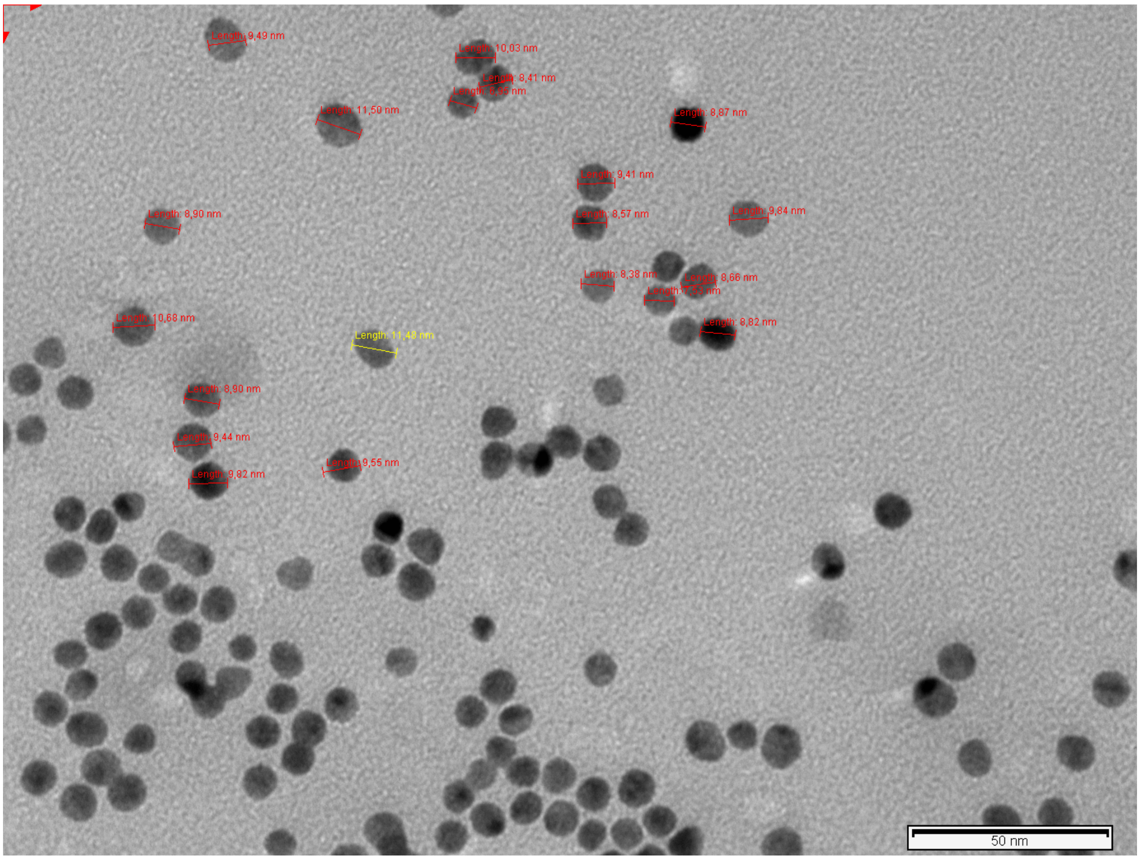

Figure 4. Characterization data and TEM images of a) bare **FeNPs**, b) **Fe@PC controls**: AgNPs after their incubation with pooled tissue extracts of disease-free individuals (controls), and c) **Fe@PC patients**: pooled tissues extracts of patients (diagnosed with FCT) in aqueous phase.

	**FeNPs**	**Fe@PC controls**	**Fe@PC patients**
1	7.33	9.64	12.28
2	9.22	12.63	8.95
3	8.79	10.21	10.99
4	7.36	12.86	11.27
5	8.99	8.40	12.19
6	7.94	14.88	14.11
7	8.15	8.95	13.92
8	8.73	12.85	10.63
9	8.62	11.17	9.30
10	7.59	11.00	10.60
11	8.41	11.07	10.71
12	8.99	12.87	10.73
13	8.78	12.56	13.63
14	8.42	14.12	14.49
15	7.27	9.69	11.31
16	7.97	13.57	13.11
17	8.78	10.25	13.76
18	8.15	11.89	12.55
19	7.55	14.43	14.24
20	8.77	8.32	11.85
21	8.87	12.10	13.07
22	9.07	11.44	10.96
23	6.07	9.99	13.48
24	8.80	10.18	11.02
25	8.15	9.60	9.62
26	6.27	12.37	11.96
27	9.82	10.60	10.21
28	7.52	9.62	9.58
29	9.42	8.84	9.92
30	8.57	10.55	11.51
31	8.36	9.86	9.96
32	8.42	8.82	10.48
33	8.15	11.49	9.58
34	7.95	14.22	13.81
35	8.59	10.94	12.17
36	7.46	13.83	11.22
37	8.36	10.99	9.96
38	8.15	10.24	10.43
39	7.34	11.77	11.22
40	8.78	12.45	12.14
Count	40	40	40
Mean	8.25	11.28	11.57
Minimum	6.27	8.32	8.95
Maximum	9.22	14.88	14.24
Standar Deviation	0.78	1.76	1.54

a) bare FeNPs in aqueous phase.

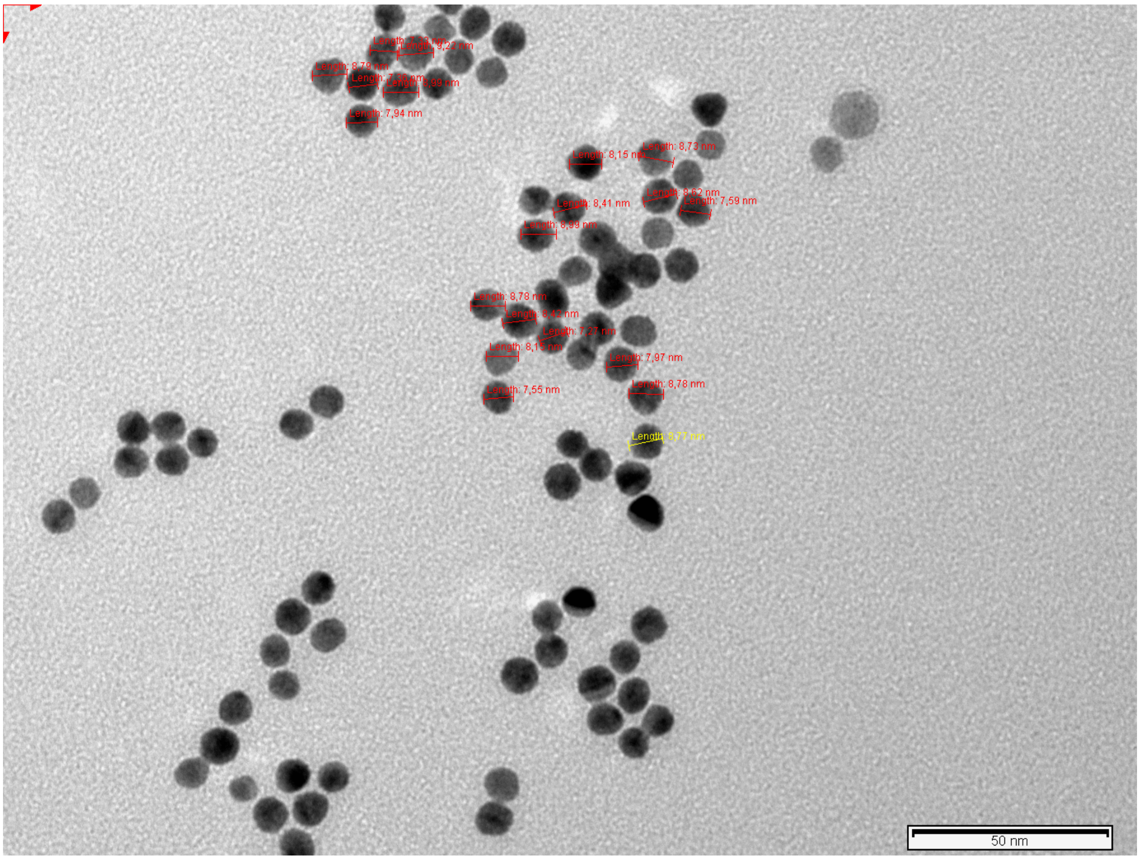

b) TEM images of AgNPs after their incubation with pooled tissue extracts of disease-free individuals (controls) in aqueous phase: FeNPs-controls.

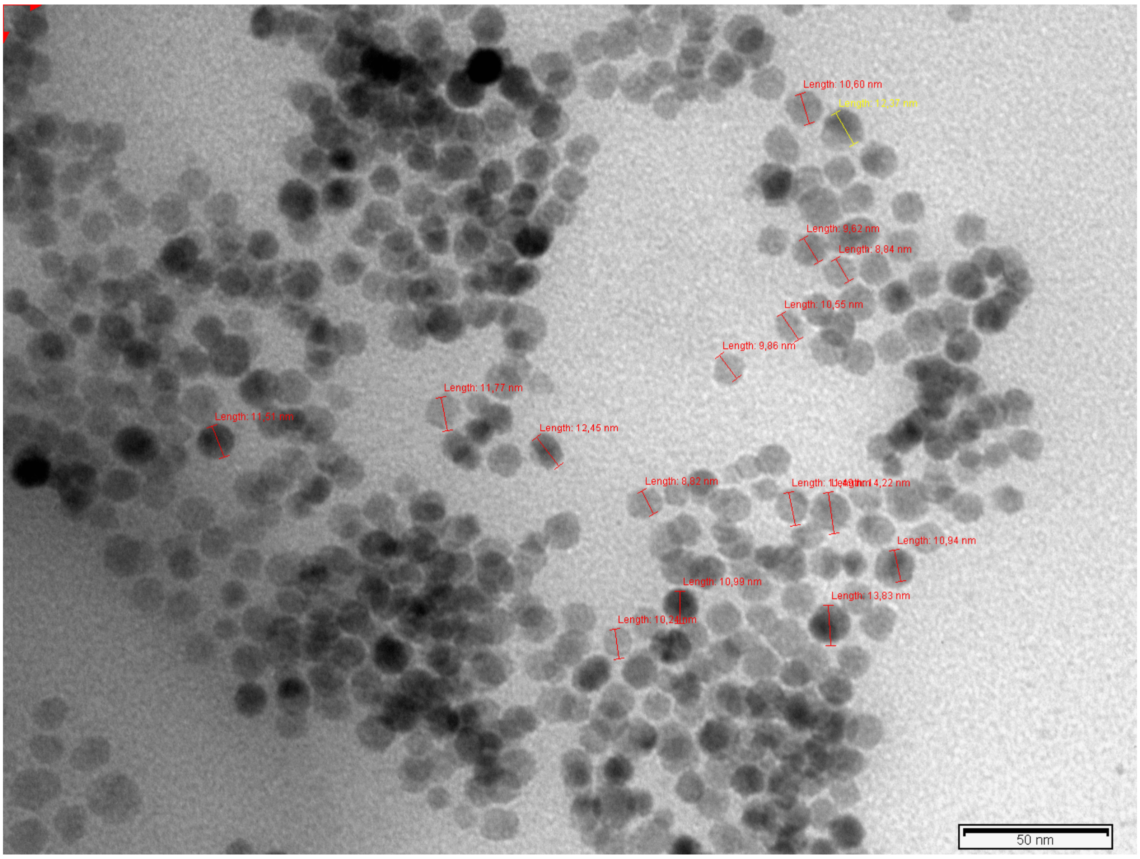

c) TEM images of FeNPs after their incubation with pooled tissue extracts of patients (diagnosed with FCT) in aqueous phase: FeNPs-patients.

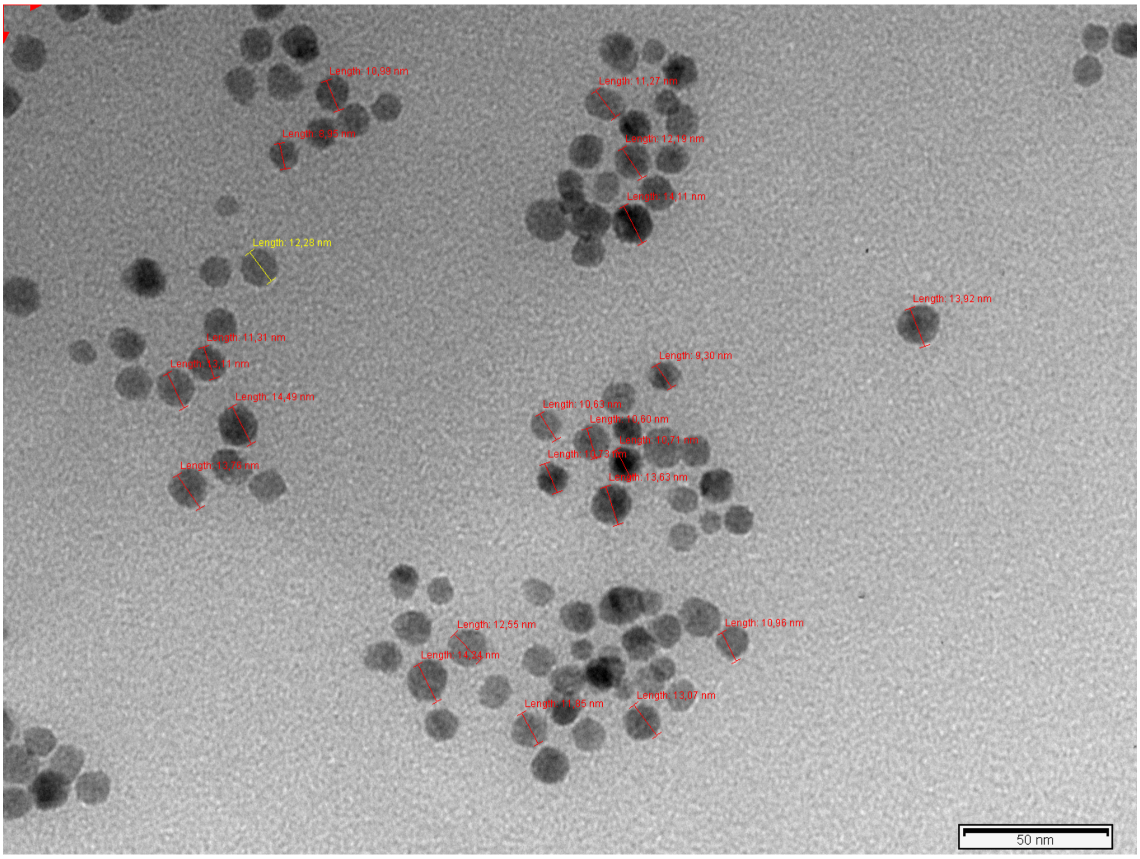

Figure S5. Venn diagrams representing the number of shared and specific molecular targets identified for the different tumor thyroid malignancies (FTA, FTC, CV-PTC and FV-PTC).

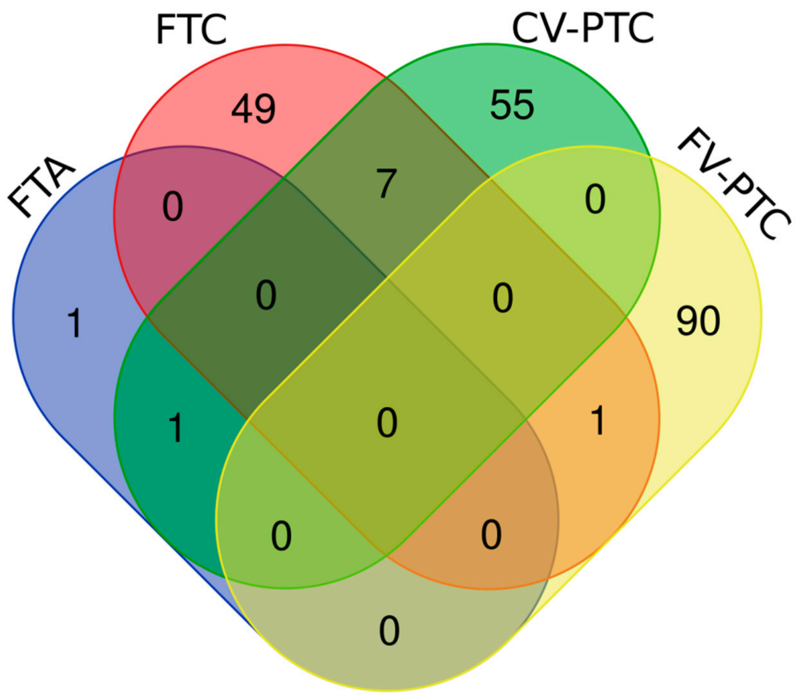

Figure S6. Classification according to the **molecular function** of the potential molecular targets identified in patients with FTA and patients with different types of thyroid cancers (FTC, CV-PTC and FV-PTC). The percentage in each category is the ratio between the number of proteins in each category and total proteins identified in that fraction.

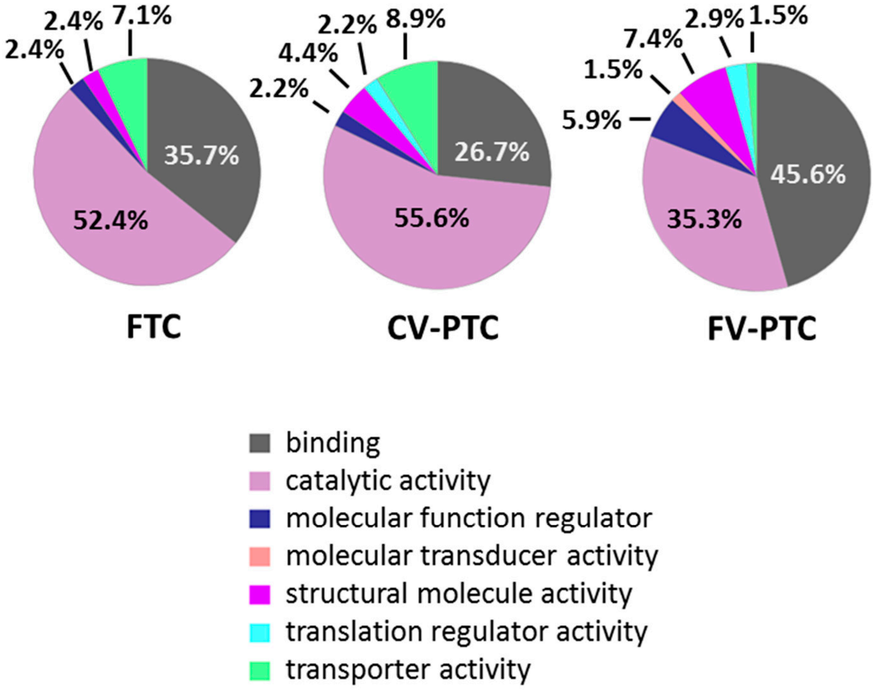

Figure S7. Classification according to the **cellular component** of the potential molecular targets in patients with FTA and patients with different types of thyroid cancers (FTC, CV-PTC and FV-PTC). The percentage in each category is the ratio between the number of proteins in each category and total proteins identified in that fraction.

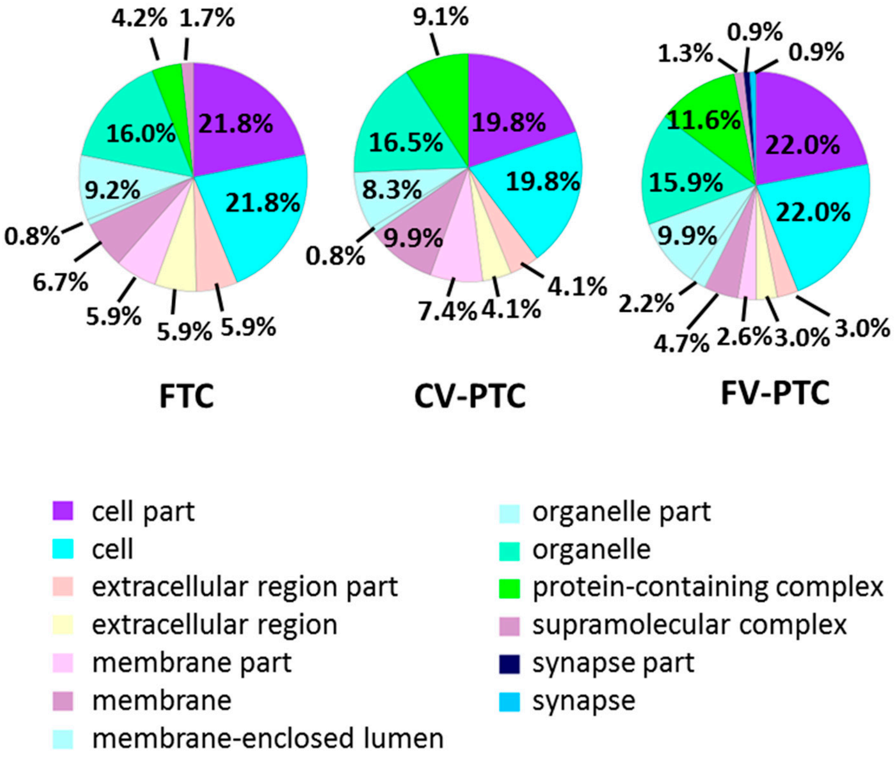

Figure S8. Classification according to the **biological process** of the potential molecular targets identified in patients with FTA and patients with different types of thyroid cancers (FTC, CV-PTC and FV-PTC). The percentage in each category is the ratio between the number of proteins in each category and total proteins identified in that fraction.

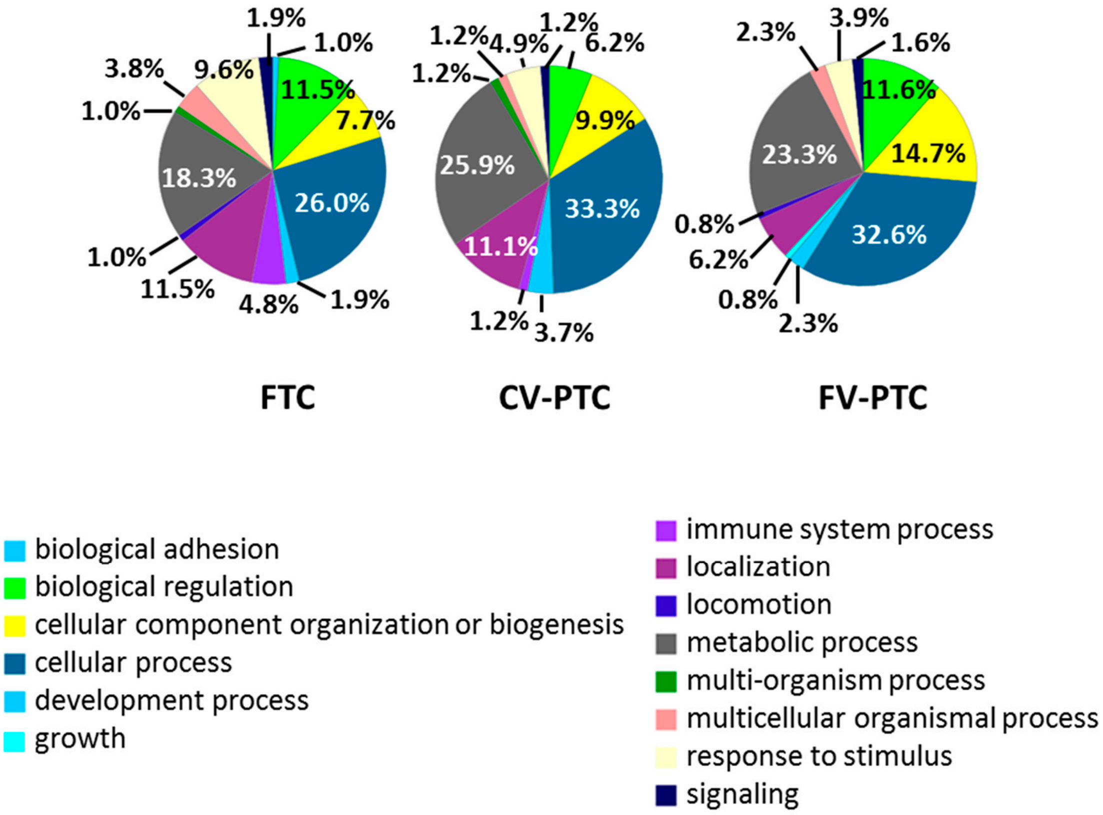

Figure S9. Significant clusters that contain terms and pathways related to the metabolic reorganization in the FTC (A), CV-PTC (B) and FV-PTC (C) PPI network analysis using the STRING v.10.0 database.

A

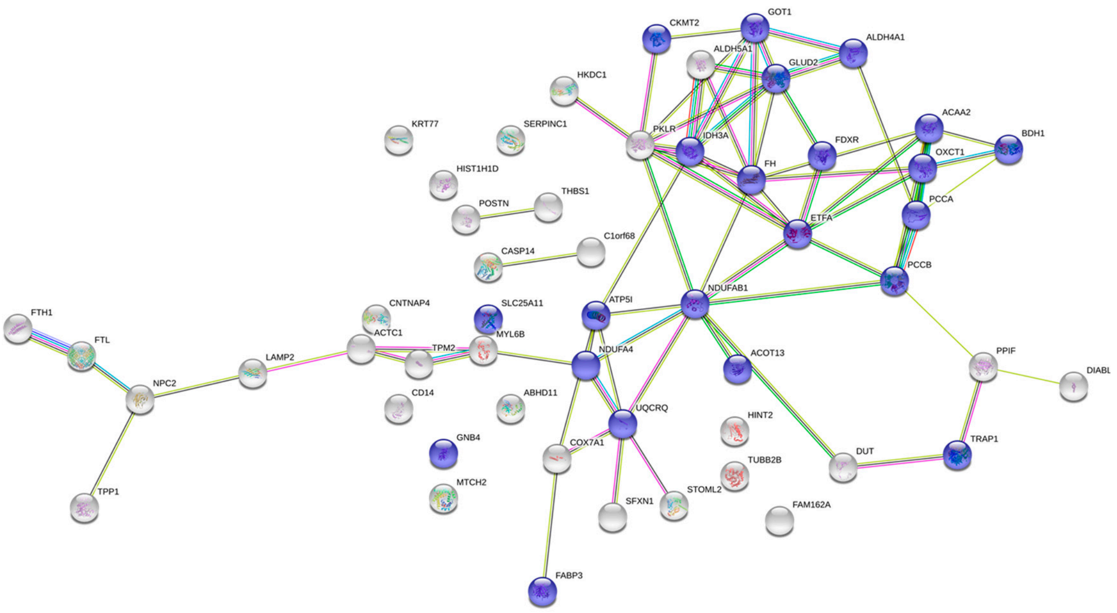

B

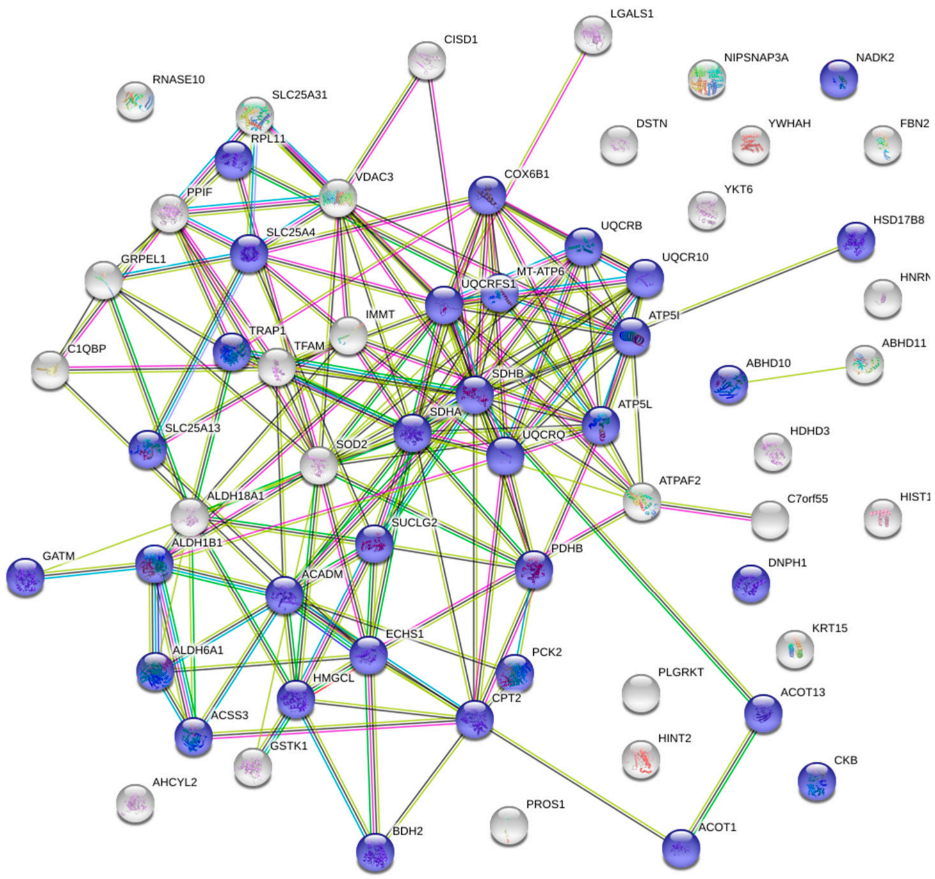

LGALS1
CISD1
NIPSNAP3A
NADK2
RNASE10
SLC25A31
RPL11
DSTN
YWHAH
FBN2
PPIF
VDAC3
COX6B1
YKT6
SLC25A4
UQCRB
HSD17B8
GRPEL1
MT-ATP6
UQCR10
HNRN
UQCRFS1
IMMT
ATP5I
TRAP1
TFAM
ABHD11
C1QBP
ABHD10
SDHB
ATP5L
SDHA
SLC25A13
UQCRQ
HDHD3
SOD2
ATPAF2
ALDH18A1
C7orf55
HIST1
SUCLG2
ALDH1B1
PDHB
GATM
DNPH1
ACADM
KRT15
ECHS1
ALDH6A1
PCK2
PLGRKT
HMGCL
CPT2
ACOT13
ACSS3
GSTK1
HINT2
CKB
AHCYL2
PROS1
BDH2
ACOT1

C

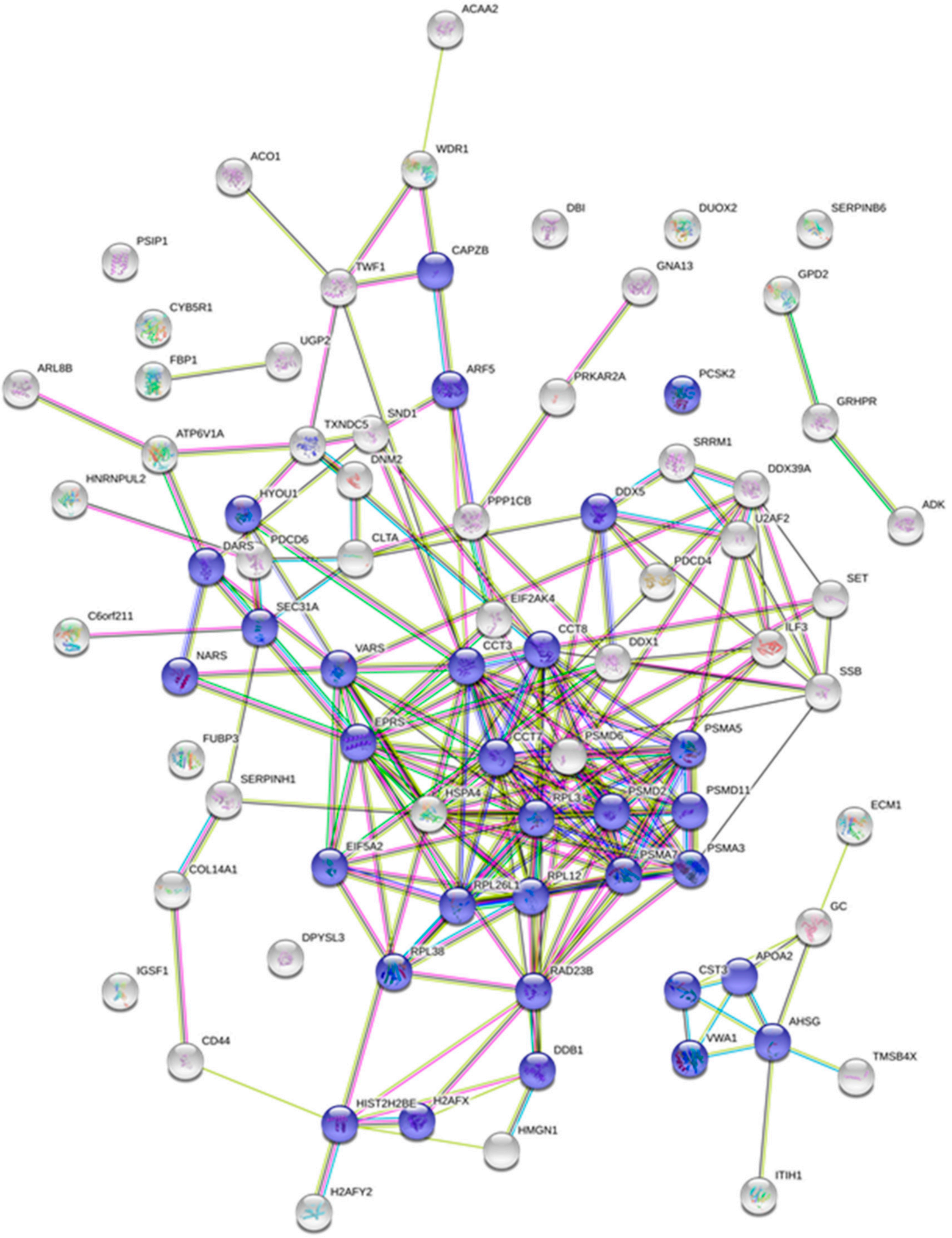
ACAA2
ACO1
WDR1
DBI
DUOX2
SERPINB6
PSIP1
CAPZB
TWF1
GNA13
GPD2
CYB5R1
UGP2
FBP1
ARL8B
ARF5
PRKAR2A
PCSK2
GRHPR
SND1
TXNDC5
ATP6V1A
DNM2
SRRM1
DDX39A
HNRNPUL2
HYOU1
PPP1CB
DDX5
ADK
U2AF2
CLTA
DARS
PDCD6
PDCD4
SET
EIF2AK4
SEC31A
C6orf211
CCT8
ILF3
DDX1
NARS
VARS
CCT3
SSB
EPRS
PSMA5
FUBP3
CCT7
PSMD6
SERPINH1
PSMD11
PSMD2
HSPA4
RPL3
ECM1
EIF5A2
PSMA7
PSMA3
COL14A1
RPL12
RPL26L1
GC
DPYSL3
RPL38
APOA2
CST3
IGSF1
RAD23B
AHSG
VWA1
DDB1
CD44
TMSB4X
HIST2H2BE
H2AFX
HMGN1
ITIH1
H2AFY2

Table 1. Shared and specific molecular targets identified for the different tumor thyroid malignancies (FTA, FTC, CV-PTC and FV-PTC).

		AgNPs				AuNPs				FeNPs			
PROTEIN	UNIPROT ID	FTA (PP1)	FTC (PP2)	CV-PTC (PP3)	FV-PTC (PP4)	FTA (PP1)	FTC (PP2)	CV-PTC (PP3)	FV-PTC (PP4)	FTA (PP1)	FTC (PP2)	CV-PTC (PP3)	FV-PTC (PP4)
14-3-3 protein eta	Q04917											x	
2'-deoxynucleoside 5'-phosphate N-hydrolase 1	O43598							x					
3-hydroxybutyrate dehydrogenase type 2	Q9BUT1							x					
3-ketoacyl-CoA thiolase. mitochondrial	P42765		x										x
60S ribosomal protein L11	P62913							x					
60S ribosomal protein L3	P39023								x				x
60S acidic ribosomal protein P0-like	Q8NHW5												x
Actin. alpha cardiac muscle 1	P68032						x						
Acyl carrier protein. mitochondrial	O14561						x						
Acyl-CoA synthetase short-chain family member 3. mitochondrial	Q9H6R3			x								x	
Acyl-CoA-binding protein	P07108								x				x
Acyl-coenzyme A thioesterase	Q86TX2											x	
Acyl-coenzyme A thioesterase 13	Q9NPJ3		x					x					
Adenosine kinase	P55263								x				
Adenosylhomocysteinase 3	Q96HN2							x					
ADP/ATP translocase 1	P12235			x									
ADP/ATP translocase 4	Q9H0C2											x	
ADP-ribosylation factor 5	P84085												x
ADP-ribosylation factor-like protein 8B	Q9NVJ2								x				
Aldehyde dehydrogenase X. mitochondrial	P30837											x	
Aldo-keto reductase family 1 member C1	Q04828					x							
Alpha-2-HS-glycoprotein	P02765								x				
Antithrombin-III	P01008		x										
Apolipoprotein A-II	P02652								x				x
Asparagine--tRNA ligase. cytoplasmic	O43776								x				
Aspartate aminotransferase. cytoplasmic	P17174		x										
Aspartate--tRNA ligase. cytoplasmic	P14868								x				
ATP-dependent RNA helicase DDX39A	O00148								x				
ATP synthase mitochondrial F1 complex assembly factor 2	Q8N5M1							x					
ATP synthase subunit a	P00846											x	
ATP synthase subunit e. mitochondrial	P56385		x									x	
ATP synthase subunit g. mitochondrial	O75964											x	
ATP-dependent RNA helicase DDX1	Q92499								x				
Bifunctional glutamate/proline--tRNA ligase	P07814								x				
Calcium-binding mitochondrial carrier protein Aralar2	Q9UJS0											x	
cAMP-dependent protein kinase type II-alpha regulatory subunit	P13861								x				
Carnitine O-palmitoyltransferase 2. mitochondrial	P23786							x					
Caspase-14	P31944						x						

CD44 antigen	P16070								x				
CDGSH iron-sulfur domain-containing protein 1	Q9NZ45							x				x	
Clathrin light chain A	P09496								x				
Collagen alpha-1(XIV) chain	Q05707								x				
Complement component 1 Q subcomponent-binding protein. mitochondrial	Q07021											x	
Contactin-associated protein-like 4	Q9C0A0						x						
Core histone macro-H2A.2	Q9P0M6								x				
Creatine kinase B-type	P12277											x	
Creatine kinase S-type. mitochondrial	P17540						x						
Cystatin-C	P01034								x				
Cytochrome b-c1 complex subunit 7	P14927											x	
Cytochrome b-c1 complex subunit 8	O14949		x									x	
Cytochrome b-c1 complex subunit 9	Q9UDW1							x					
Cytochrome b-c1 complex subunit Rieske. mitochondrial	P47985							x				x	
Cytochrome c oxidase subunit 6B1	P14854											x	
Cytochrome c oxidase subunit 7A1. mitochondrial	P24310						x						
Cytochrome c oxidase subunit NDUFA4	O00483		x										
Cytoplasmic aconitate hydratase	P21399								x				
Damage-control phosphatase ARMT1	Q9H993								x				
D-beta-hydroxybutyrate dehydrogenase. mitochondrial	Q02338		x										
Delta-1-pyrroline-5-carboxylate dehydrogenase. mitochondrial	P30038						x				x		
Delta-1-pyrroline-5-carboxylate synthase	P54886							x					
Deoxyuridine 5'-triphosphate nucleotidohydrolase. mitochondrial	P33316						x						
Destrin	P60981							x					
DNA damage-binding protein 1	Q16531								x				
Diablo homolog. mitochondrial	Q9NR28		x				x						
Dihydropyrimidinase-related protein 3	Q14195								x				x
Dual oxidase 2	Q9NRD8								x				
Dynamin-2	P50570								x				
Electron transfer flavoprotein subunit alpha. mitochondrial	P13804		x										
eIF-2-alpha kinase GCN2	Q9P2K8								x				
Enoyl-CoA hydratase. mitochondrial	P30084											x	
Estradiol 17-beta-dehydrogenase 8	Q92506							x					
Eukaryotic translation initiation factor 5A-2	Q9GZV4												x
Extracellular matrix protein 1	Q16610								x				
F-actin-capping protein subunit beta	P47756								x				
Far upstream element-binding protein 3	Q96I24								x				
Fatty acid-binding protein. heart	P05413		x				x				x		
Ferritin heavy chain	P02794		x				x				x		
Ferritin light chain	P02792		x				x				x		
Fibrillin-2	P35556							x					

Fructose-1.6-bisphosphatase 1	P09467							x					
Fumarate hydratase. mitochondrial	P07954		x										
Galectin-1	P09382							x					
Glutamate dehydrogenase 2. mitochondrial	P49448						x						
Glutathione S-transferase kappa 1	Q9Y2Q3							x					
Glycerol-3-phosphate dehydrogenase. mitochondrial	P43304								x				
Glycine amidinotransferase. mitochondrial	P50440			x								x	
Glyoxylate reductase/hydroxypyruvate reductase	Q9UBQ7								x				
GrpE protein homolog 1. mitochondrial	Q9HAV7							x					
Guanine nucleotide-binding protein subunit alpha-13	Q14344								x				
Guanine nucleotide-binding protein subunit beta-4	Q9HAV0						x						
Haloacid dehalogenase-like hydrolase domain-containing protein 3	Q9BSH5							x					
Heat shock 70 kDa protein 4	P34932								x				
Heat shock protein 75 kDa. mitochondrial	Q12931		x									x	
Heterogeneous nuclear ribonucleoprotein C-like 2	B2RXH8			x		x							
Heterogeneous nuclear ribonucleoprotein U-like protein 2	Q1KMD3								x				x
Hexokinase HKDC1	Q2TB90		x				x				x		
Histidine triad nucleotide-binding protein 2. mitochondrial	Q9BX68		x					x				x	
Histone H1.3	P16402		x								x		
Histone H2AX	P16104												x
Histone H2B type 1-N	Q99877											x	
Histone H2B type 3-B	Q8N257								x				
Hydroxymethylglutaryl-CoA lyase. mitochondrial	P35914							x					
Hypoxia up-regulated protein 1	Q9Y4L1												x
Immunoglobulin heavy constant alpha 2	P01877											x	
Immunoglobulin kappa variable 2D-28	P01615						x						
Immunoglobulin kappa variable 3-11	P04433						x						
Immunoglobulin kappa variable 3-15	P01624										x		
Immunoglobulin kappa variable 3-20	P01619										x		
Immunoglobulin superfamily member 1	Q8N6C5				x				x				x
Inactive ribonuclease-like protein 10	Q5GAN6							x					
Inter-alpha-trypsin inhibitor heavy chain H1	P19827								x				
Interleukin enhancer-binding factor 3	Q12906												x
Isocitrate dehydrogenase [NAD] subunit alpha. mitochondrial	P50213		x										
Keratin. type I cytoskeletal 15	P19012			x									
Keratin. type II cytoskeletal 1b	Q7Z794						x						
Lupus La protein	P05455								x				
Lysosome-associated membrane glycoprotein 2	P13473						x						
Medium-chain specific acyl-CoA dehydrogenase. mitochondrial	P11310			x								x	

Methylmalonate-semialdehyde dehydrogenase [acylating]. mitochondrial	Q02252											x	
MICOS complex subunit MIC60	Q16891											x	
Mitochondrial 2-oxoglutarate/malate carrier protein	Q02978		x										
Mitochondrial carrier homolog 2	Q9Y6C9		x										
Monocyte differentiation antigen CD14	P08571						x						
Mycophenolic acid acyl-glucuronide esterase. mitochondrial	Q9NUJ1											x	
Myosin light chain 6B	P14649		x										
NAD kinase 2. mitochondrial	Q4G0N4							x					
NADH-cytochrome b5 reductase 1	Q9UHQ9								x				
NADPH:adrenodoxin oxidoreductase. mitochondrial	P22570						x						
Neuroendocrine convertase 2	P16519								x				x
Non-histone chromosomal protein HMG-14	P05114								x				
NPC intracellular cholesterol transporter 2	P61916		x										
PC4 and SFRS1-interacting protein	O75475								x				
Peptidyl-prolyl cis-trans isomerase F. mitochondrial	P30405		x									x	
Periostin	Q15063		x								x		
Phosphoenolpyruvate carboxykinase [GTP]. mitochondrial	Q16822											x	
Plasminogen receptor (KT)	Q9HBL7							x					
Probable ATP-dependent RNA helicase DDX5	P17844								x				
Programmed cell death protein 4	Q53EL6								x				
Programmed cell death protein 6	O75340								x				
Propionyl-CoA carboxylase alpha chain. mitochondrial	P05165		x										
Propionyl-CoA carboxylase beta chain. mitochondrial	P05166						x						
26S proteasome non-ATPase regulatory subunit 2	Q13200								x				
26S proteasome non-ATPase regulatory subunit 6	Q15008								x				
26S proteasome non-ATPase regulatory subunit 11	O00231								x				
Proteasome subunit alpha type-3	P25788								x				
Proteasome subunit alpha type-5	P28066								x				x
Proteasome subunit alpha type-7	O14818								x				
Protein ABHD11	Q8NFV4		x									x	
Protein FAM162A	Q96A26						x						
Protein FMC1 homolog	Q96HJ9							x					
Protein NipSnap homolog 3A	Q9UFN0							x					
Protein SET	Q01105												x
Protein transport protein Sec31A	O94979								x				
Pyruvate dehydrogenase E1 component subunit beta. mitochondrial	P11177											x	
Pyruvate kinase PKLR	P30613		x										
60S ribosomal protein L12	P30050												x
60S ribosomal protein L26-like 1	Q9UNX3								x				

60S ribosomal protein L38	P63173								x				
Serine/arginine repetitive matrix protein 1	Q8IYB3								x				
Serine/threonine-protein phosphatase PP1-beta catalytic subunit	P62140								x				
Serpin B6	P35237								x				
Serpin H1	P50454								x				
Sideroflexin-1	Q9H9B4		x				x						
Skin-specific protein 32	Q5T750						x						
Splicing factor U2AF 65 kDa subunit	P26368								x				
Staphylococcal nuclease domain-containing protein 1	Q7KZF4								x				
Stomatin-like protein 2. mitochondrial	Q9UJZ1						x						
Succinate dehydrogenase [ubiquinone] flavoprotein subunit. mitochondrial	P31040											x	
Succinate dehydrogenase [ubiquinone] iron-sulfur subunit. mitochondrial	P21912							x					
Succinate--CoA ligase [GDP-forming] subunit beta. mitochondrial	Q96I99											x	
Succinate-semialdehyde dehydrogenase. mitochondrial	P51649		x										
Succinyl-CoA:3-ketoacid coenzyme A transferase 1. mitochondrial	P55809		x										
Superoxide dismutase [Mn]. mitochondrial	P04179											x	
Synaptobrevin homolog YKT6	O15498											x	
T-complex protein 1 subunit eta	Q99832								x				
T-complex protein 1 subunit gamma	P49368								x				
T-complex protein 1 subunit theta	P50990								x				
Thioredoxin domain-containing protein 5	Q8NBS9								x				
Thrombospondin-1	P07996						x						
Thymosin beta-4	P62328								x				
Transcription factor A. mitochondrial	Q00059											x	
Tripeptidyl-peptidase 1	O14773										x		
Tropomyosin beta chain	P07951						x						
Tubulin beta-2B chain	Q9BVA1		x										
Twinfilin-1	Q12792								x				
UTP--glucose-1-phosphate uridylyltransferase	Q16851								x				
UV excision repair protein RAD23 homolog B	P54727								x				
Valine--tRNA ligase	P26640								x				
Vitamin D-binding protein	P02774								x				
Vitamin K-dependent protein S	P07225							x					
Voltage-dependent anion-selective channel protein 3	Q9Y277							x				x	
von Willebrand factor A domain-containing protein 1	Q6PCB0								x				
V-type proton ATPase catalytic subunit A	P38606								x				
WD repeat-containing protein 1	O75083												x

Table 2. Specific molecular targets identified for the different thyroid malignancies (FTA, FTC, CV-PTC and FV-PTC). Proteins in bold were indetified in more than one subtype.

FTA<br>*Follicular thyroid adenoma*	CV-PTC<br>*Classical variant of papillary thyroid carcinoma*	FV-PTC<br>*Follicular variant of papillary thyroid carcinoma*
AuNPs (n = 2)	*AgNPs-FeNPs (n = 3)*	*AgNPs-AuNPs-FeNPs (n = 1)*
Aldo-keto reductase family 1 member C1 (AKR1C1)	Acyl-CoA synthetetase short-chain family member 3, mitocondrial (ACSS3)	Immunoglobulin superfamily member 1 (IGSF1)
**Heterogeneous nuclear ribonucleoprotein C-like 2 (HNRNPCL2)**	Glycine amidinotransferase, mitocondrial (GATM)	*AuNPs-FeNPs (n = 7)*
FTC<br>*Follicular thyroid carcinoma*	Medium-chain specific acyl-CoA dehydrogenase, mitocondrial (ACADM)	Acyl-CoA-binding protein (DBI)
AgNPs-AuNPs-FeNPs (n = 4)	*AuNPs-FeNPs (n = 4)*	Apolipoprotein A-II (APOA2)
Fatty acid-binding protein, heart (FABP3)	CDGSH iron-sulfur domain-containing protein 1 (CISD1)	Dihydropyrimidinase-related protein 3 (DPYSL3)
Ferritin heavy chain (FTH1)	Cytochrome b-c1 complex subunit Rieske, mitocondrial (UQCRFS1)	Heterogeneous nuclear ribonucleoprotein U-like protein 2 (HNRNPUL2)
Ferritin light chain (FTL)	**Histidine triad nucleotide-binding protein 2, mitocondrial (HINT2)**	Neuroendocrine convertase 2 (PCSK2)
Hexokinase HKDC1 (HKDC1)	Voltage-dependent anion-selective channel protein 3 (VDAC3)	Proteasome subunit alpha type-5 (PSMA5)
AgNPs-AuNPs (n = 2)	*AgNPs (n = 3)*	60S ribosomal protein L3 (RPL3)
Diablo homolog, mitocondrial (DIABLO)	ADP/ATP translocase 1 (SLC25A4)	*AuNPs (n = 73)*
Sideroflexin-1 (SFXN1)	**Heterogeneous nuclear ribonucleoprotein C-like 2 (HNRNPCL2)**	Adenosine kinase (ADK)
AgNPs-FeNPs (n = 2)	Keratin, type I cytoskeletal 15 (KRT15)	ADP-ribosylation factor-like protein 8B (ARL8B)
Histone H1.3 (H1-3)	*AuNPs (n = 24)*	Alpha-2-HS-glycoprotein (AHSG)
		Aminopeptidase B (RNPEP)
Periostin (POSTN)	**Acyl-coenzyme A thioesterase 13 (ACOT13)**	Asparagine--tRNA ligase, cytoplasmic (NARS1)
AuNPs-FeNPs (n = 1)	Adenosylhomocysteinase 3 (AHCYL2)	Aspartate--tRNA ligase, cytoplasmic (DARS1)
Delta-1-pyrroline-5-carboxylate dehydrogenase, mitocondrial (ALDH4A1)	ATP synthase mitochondrial F1 complex assembly factor 2 (ATPAF2)	ATP-dependent RNA helicase DDX1 (DDX1)
AgNPs (n = 24)	Carnitine O-palmitoyltransferase 2, mitocondrial (CPT2)	ATP-dependent RNA helicase DDX39A (DDX39A)
**Acyl-coenzyme A thioesterase 13 (ACOT13)**	Cytochrome b-c1 complex subunit 9 (UQCR10)	Bifunctional glutamate/proline--tRNA ligase (EPRS1)
Antithrombin-III (SERPINC1)	Delta-1-pyrroline-5-carboxylate synthase (ALDH18A1)	cAMP-dependent protein kinase type II-alpha regulatory subunit (PRKAR2A)
Aspartate aminotransferase, cytoplasmic (GOT1)	2'-deoxynucleoside 5'-phosphate N-hydrolase 1 (DNPH1)	CD44 antigen (CD44)
**ATP synthase subunit e, mitocondrial (ATP5ME)**	Destrin (DSTN)	Clathrin light chain A (CLTA)
**Cytochrome b-c1 complex subunit 8 (UQCRQ)**	Estradiol 17-beta-dehydrogenase 8 (HSD17B8)	Collagen alpha-1(XIV) chain (COL14A1)
Cytochrome c oxidase subunit NDUFA4 (NDUFA4)	Fibrillin-2 (FBN2)	Core histone macro-H2A.2 (MACROH2A2)
D-beta-hydroxybutyrate dehydrogenase, mitocondrial (BDH1)	Galectin-1 (LGALS1)	Cystatin-C (CST3)
Electron transfer flavoprotein subunit alpha, mitocondrial (ETFA)	Glutathione S-transferase kappa 1 (GSTK1)	Cytoplasmic aconitate hydratase (ACO1)
Fumarate hydratase, mitocondrial (FH)	GrpE protein homolog 1, mitocondrial (GRPEL1)	Damage-control phosphatase ARMT1 (ARMT1)
**Heat shock protein 75 kDa, mitocondrial (TRAP1)**	Haloacid dehalogenase-like hydrolase domain-containing protein 3 (HDHD3)	DNA damage-binding protein 1 (DDB1)
		Dual oxidase 2 (DUOX2)
**Histidine triad nucleotide-binding protein 2, mitocondrial (HINT2)**	3-hydroxybutyrate dehydrogenase type 2 (BDH2)	Dynamin-2 (DNM2)
		EF-hand domain-containing protein D2 (EFHD2)
Isocitrate dehydrogenase [NAD] subunit alpha, mitocondrial (IDH3A)	Hydroxymethylglutaryl-CoA lyase, mitocondrial (HMGCL)	eIF-2-alpha kinase GCN2 (EIF2AK4)
**3-ketoacyl-CoA thiolase, mitocondrial (ACAA2)**	Inactive ribonuclease-like protein 10 (RNASE10)	Extracellular matrix protein 1 (ECM1)
Mitochondrial carrier homolog 2 (MTCH2)	NAD kinase 2, mitocondrial (NADK2)	F-actin-capping protein subunit beta (CAPZB)
Mitochondrial 2-oxoglutarate/malate carrier protein (SLC25A11)	Plasminogen receptor (KT) (PLGRKT)	Far upstream element-binding protein 3 (FUBP3)
		Flotillin-1 (FLOT1)
Myosin light chain 6B (MYL6B)	Protein FMC1 homolog (FMC1)	Fructose-1,6-bisphosphatase 1 (FBP1)
NPC intracellular cholesterol transporter 2 (NPC2)	Protein NipSnap homolog 3A (NIPSNAP3A)	Glycerol-3-phosphate dehydrogenase, mitocondrial (GPD2)
**Peptidyl-prolyl cis-trans isomerase F, mitocondrial (PPIF)**	60S ribosomal protein L11 (RPL11)	Glyoxylate reductase/hydroxypyruvate reductase (GRHPR)
Propionyl-CoA carboxylase alpha chain, mitocondrial (PCCA)	Succinate dehydrogenase [ubiquinone] iron-sulfur subunit, mitocondrial (SDHB)	Guanine nucleotide-binding protein subunit alpha-13 (GNA13)
**Protein ABHD11 (ABHD11)**	Vitamin K-dependent protein S (PROS1)	Heat shock 70 kDa protein 4 (HSPA4)

Pyruvate kinase PKLR (PKLR)	*FeNPs (n = 29)*	Histone H2B type 3-B (H2BU1)
Succinate-semialdehyde dehydrogenase, mitochondrial (ALDH5A1)	Acyl-coenzyme A thioesterase 1 (ACOT1)	Inter-alpha-trypsin inhibitor heavy chain H1 (ITIH1)
		Leucine-rich repeat-containing protein 47 (LRRC47)
Succinyl-CoA:3-ketoacid coenzyme A transferase 1, mitochondrial (OXCT1)	ADP/ATP translocase 4 (SLC25A31)	Lupus La protein (SSB)
Tubulin beta-2B chain (TUBB2B)	Aldehyde dehydrogenase X, mitochondrial (ALDH1B1)	NADH-cytochrome b5 reductase 1 (CYB5R1)
		N(G),N(G)-dimethylarginine dimethylaminohydrolase 1 (DDAH1)
AuNPs (n = 21)	ATP synthase subunit a (MT-ATP6)	Non-histone chromosomal protein HMG-14 (HMGN1)
Actin, alpha cardiac muscle 1 (ACTC1)	**ATP synthase subunit e, mitochondrial (ATP5ME)**	PC4 and SFRS1-interacting protein (PSIP1)
		Plastin-1 (PLS1)
Acyl carrier protein, mitochondrial (NDUFAB1)	ATP synthase subunit g, mitochondrial (ATP5MG)	Probable ATP-dependent RNA helicase DDX5 (DDX5)
Caspase-14 (CASP14)	Calcium-binding mitochondrial carrier protein Aralar2 (SLC25A13)	Programmed cell death protein 4 (PDCD4)
Contactin-associated protein-like 4 (CNTNAP4)	Complement component 1 Q subcomponent-binding protein, mitochondrial (C1QBP)	Programmed cell death protein 6 (PDCD6)
Creatine kinase S-type, mitochondrial (CKMT2)	Creatine kinase B-type (CKB)	Proteasome subunit alpha type-3 (PSMA3)
		Proteasome subunit alpha type-6 (PSMA6)
Cytochrome c oxidase subunit 7A1, mitochondrial (COX7A1)	Cytochrome b-c1 complex subunit 7 (UQCRB)	Proteasome subunit alpha type-7 (PSMA7)
Deoxyuridine 5'-triphosphate nucleotidohydrolase, mitochondrial (DUT)	**Cytochrome b-c1 complex subunit 8 (UQCRQ)**	Protein transport protein Sec31A (SEC31A)
Glutamate dehydrogenase 2, mitochondrial (GLUD2)	Cytochrome c oxidase subunit 6B1 (COX6B1)	26S proteasome non-ATPase regulatory subunit 2 (PSMD2)
Guanine nucleotide-binding protein subunit beta-4 (GNB4)	Enoyl-CoA hydratase, mitochondrial (ECHS1)	26S proteasome non-ATPase regulatory subunit 6 (PSMD6)
Immunoglobulin kappa variable 2D-28 (IGKV2D-28)	**Heat shock protein 75 kDa, mitochondrial (TRAP1)**	26S proteasome non-ATPase regulatory subunit 11 (PSMD11)
		26S proteasome non-ATPase regulatory subunit 12 (PSMD12)
		Rho GDP-dissociation inhibitor 1 (ARHGDIA)
Immunoglobulin kappa variable 3-11 (IGKV3-11)	Histone H2B type 1-N (H2BC15)	60S ribosomal protein L26-like 1 (RPL26L1)
Keratin, type II cytoskeletal 1b (KRT77)	Immunoglobulin heavy constant alpha 2 (IGHA2)	60S ribosomal protein L38 (RPL38)
Lysosome-associated membrane glycoprotein 2 (LAMP2)	Methylmalonate-semialdehyde dehydrogenase [acylating], mitochondrial (ALDH6A1)	Serine/arginine repetitive matrix protein 1 (SRRM1)
Monocyte differentiation antigen CD14 (CD14)	MICOS complex subunit MIC60 (IMMT)	Serine/threonine-protein phosphatase PP1-beta catalytic subunit (PPP1CB)
NADPH:adrenodoxin oxidoreductase, mitochondrial (FDXR)	Mycophenolic acid acyl-glucuronide esterase, mitochondrial (ABHD10)	Serpin B6 (SERPINB6)
Propionyl-CoA carboxylase beta chain, mitochondrial (PCCB)	**Peptidyl-prolyl cis-trans isomerase F, mitochondrial (PPIF)**	Serpin H1 (SERPINH1)
Protein FAM162A (FAM162A)	Phosphoenolpyruvate carboxykinase [GTP], mitochondrial (PCK2)	Splicing factor U2AF 65 kDa subunit (U2AF2)
Skin-specific protein 32 (XP32)	**Protein ABHD11 (ABHD11)**	Staphylococcal nuclease domain-containing protein 1 (SND1)
Stomatin-like protein 2, mitochondrial (STOML2)	14-3-3 protein eta (YWHAH)	T-complex protein 1 subunit eta (Q99832)
Thrombospondin-1 (THBS1)	Pyruvate dehydrogenase E1 component subunit beta, mitochondrial (PDHB)	T-complex protein 1 subunit gamma (CCT3)
Tropomyosin beta chain (TPM2)	Succinate--CoA ligase [GDP-forming] subunit beta, mitochondrial (SUCLG2)	T-complex protein 1 subunit theta (CCT8)
FeNPs (n = 3)	Succinate dehydrogenase [ubiquinone] flavoprotein subunit, mitochondrial (SDHA)	Thioredoxin domain-containing protein 5 (TXNDC5)
Immunoglobulin kappa variable 3-15 (IGKV3-15)	Superoxide dismutase [Mn], mitochondrial (SOD2)	Thymosin beta-4 (TMSB4X)
		Tryptophan--tRNA ligase, cytoplasmic (WARS1)
Immunoglobulin kappa variable 3-20 (IGKV3-20)	Synaptobrevin homolog YKT6 (YKT6)	Twinfilin-1 (TWF1)
Tripeptidyl-peptidase 1 (TPP1)	Transcription factor A, mitochondrial (TFAM)	UTP--glucose-1-phosphate uridylyltransferase (UGP2)
		UV excision repair protein RAD23 homolog B (RAD23B)
		Valine--tRNA ligase (VARS1)
		Vitamin D-binding protein (GC)
		von Willebrand factor A domain-containing protein 1 (VWA1)
		V-type proton ATPase catalytic subunit A (ATP6V1A)

FeNPs (n = 10)
ADP-ribosylation factor 5 (ARF5)
60S acidic ribosomal protein P0-like (RPLP0P6)
Eukaryotic translation initiation factor 5A-2 (EIF5A2)
Histone H2AX (H2AX)
Hypoxia up-regulated protein 1 (HYOU1)
Interleukin enhancer-binding factor 3 (ILF3)
**3-ketoacyl-CoA thiolase, mitocondrial (ACAA2)**
Protein SET (SET)
60S ribosomal protein L12 (RPL12)
WD repeat-containing protein 1 (WDR1)
